# Polyethylenimine Carriers for Drug and Gene Delivery

**DOI:** 10.3390/polym17152150

**Published:** 2025-08-06

**Authors:** Ahmed Ismail, Shih-Feng Chou

**Affiliations:** 1 Department of Mechanical Engineering, College of Engineering, The University of Texas at Tyler, Tyler, TX 75799, USA; aismail2@patriots.uttyler.edu; 2 Advanced Materials and Manufacturing Institute, College of Engineering, The University of Texas at Tyler, Tyler, TX 75799, USA

**Keywords:** polyethylenimine, polymerization, drug carriers, structure–property relationships, cytotoxicity, gene therapy, drug delivery

## Abstract

Polyethylenimine (PEI) is a cationic polymer with a high density of amine groups suitable for strong electrostatic interactions with biological molecules to preserve their bioactivities during encapsulation and after delivery for biomedical applications. This review provides a comprehensive overview of PEI as a drug and gene carrier, describing its polymerization methods in both linear and branched forms while highlighting the processing methods to manufacture PEIs into drug carriers, such as nanoparticles, coatings, nanofibers, hydrogels, and films. These various PEI carriers enable applications in non-viral gene and small molecule drug deliveries. The structure–property relationships of PEI carriers are discussed with emphasis on how molecular weights, branching degrees, and surface modifications of PEI carriers impact biocompatibility, transfection efficiency, and cellular interactions. While PEI offers remarkable potential for drug and gene delivery, its clinical translation remains limited by challenges, including cytotoxicity, non-degradability, and serum instability. Our aim is to provide an understanding of PEI and the structure–property relationships of its carrier forms to inform future research directions that may enable safe and effective clinical use of PEI carriers for drug and gene delivery.

## 1. Introduction

Modern biomedical engineering requires the development of drug and gene delivery systems to overcome limitations associated with conventional formulations. Drug delivery has transformed treatment by enabling site-specific administration, prolonged circulation, and protection of fragile drugs. Common carriers include liposomes, micelles, dendrimers, solid lipid nanoparticles, hydrogels, and polymeric or inorganic nanoparticles [1]. These drug carriers are engineered to improve solubility, minimize off-target effects, and regulate drug releases. Despite all these improvements, drug deliveries from polymer carriers still face challenges, particularly as the majority of uncontrolled or fast drug release is associated with them [2]. Other related issues are low drug-loading capacity [3] as well as toxicity concerns due to the non-degradability of polymer carriers [4]. Therefore, current research in drug delivery focuses on the performance and safety of polymer carriers, which drives innovations in their synthesis and design for more biocompatible, higher-drug-loading, and controlled release carriers [5].

Gene delivery introduces nucleic acids, such as plasmid DNA, siRNA, and mRNA, into target cells for a therapeutic effect. Polymer carriers for gene delivery fall into two major categories: viral vectors (e.g., lentivirus, adenovirus) and non-viral systems, including cationic liposomes, dendrimers, polyethylenimine (PEI), chitosan, poly(lactic-*co*-glycolic acid) (PLGA), and lipid–polymer hybrids [6]. Due to their lower immunogenicity and easier production, non-viral vectors have drawn increasing attention. However, the clinical translations from laboratories of these non-viral vectors are hindered by inefficient endosomal escape [7], cytotoxicity, and non-biodegradability of cationic polymer carriers [8]. Their low transfection efficiencies limit their therapeutic benefit; in the meantime, balancing their biocompatibility with therapeutic performance remains challenging [9].

Among the diverse polymeric materials investigated for therapeutic delivery, polyethylenimine (PEI) has garnered particular interest due to its remarkable ability to condense nucleic acids into stable complexes, thereby enhancing cellular uptake and facilitating endosomal escape [10]. Its strong cationic character enables robust electrostatic interactions with negatively charged biomolecules and cellular membranes, making it especially suitable for gene delivery applications [11]. PEI’s structure is also highly tunable, allowing modifications to enhance its biocompatibility, targeting, and delivery efficiency across therapeutic platforms [12]. Although concerns have historically been raised regarding its cytotoxicity and limited biodegradability, recent advances in its formulation and structural design have helped address these issues, further solidifying PEI’s position as a cornerstone material in the design of next-generation non-viral gene delivery systems [11].

This review presents an overview of PEI as a drug carrier for encapsulations and delivery of small molecule drugs and non-viral gene vectors in biomedical applications. We discuss the polymerization of PEI, followed by the fabrication of PEI into structural forms of nanoparticles, coatings, nanofibers, hydrogels, and films to further enable it to carry drugs/genes. In addition, we review the structure–property relationships of these PEI drug carriers, where each carrier form offers distinct advantages in terms of encapsulation, targeting specificity, and controlled release. While PEI is the frontrunner as a polymer carrier for gene delivery, there are still limitations and obstacles to overcome before its transition to clinical studies in humans. In general, this review addresses the scientific understanding of PEI for carrier materials with an emphasis on PEI carriers in biomedical applications for drug and gene delivery.

## 2. Polymerization of Polyethylenimine (PEI)

PEI is primarily polymerized through the ring-opening polymerization of aziridine, producing either linear PEI (LPEI) or branched PEI (BPEI) depending on the reaction conditions and catalysts, as well as the stoichiometric ratios of the reactants. In the case of LPEI, polymerization typically involves *N*-substituted polyaziridine or 2-oxazolines, such as 2-ethyl-2-oxazoline, followed by hydrolysis to yield the linear polymer [13]. Polymer architecture is dependent on the choice of monomer and polymerization conditions, allowing for controlled molecular weights and degrees of polymerization. LPEI is easier to polymerize and has a lower molecular weight compared to branched PEI [14]. Conversely, BPEI synthesis requires strict control to achieve specific branching patterns; nevertheless, BPEI holds the dominant position in the market, accounting for approximately 73.40% of the total PEI revenue as of 2022, while LPEI comprises the remainder [15]. Table 1 illustrates the comparative performance of LPEI and BPEI in gene and drug delivery as well as their co-delivery applications.

### 2.1. Polymerization of Linear PEI

LPEI is a synthetic polyamine polymer composed entirely of secondary amine groups arranged in a straight-chain structure. Mees and Hoogenboom [13] synthesized LPEI cationically by polymerizing 2-oxazoline or 2-methyloxazoline in dimethylformamide followed by hydrolysis of the product under alkaline conditions. The produced LPEI had a homogeneous molecular structure preferred for applications in adhesives, coatings, and targeted gene delivery systems, where controlled behavior and decreased cytotoxicity are crucial. In addition, LPEI is extremely useful for creating functional materials that need precise chemical modification because of its well-defined structure [12].

**Figure 1:**
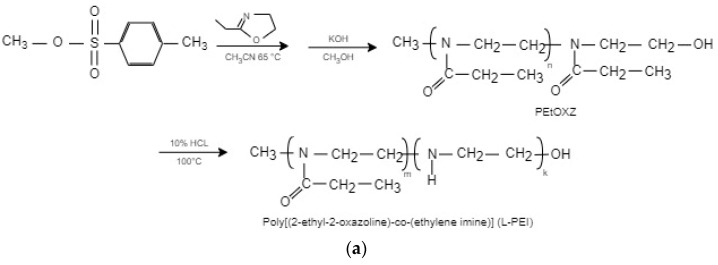

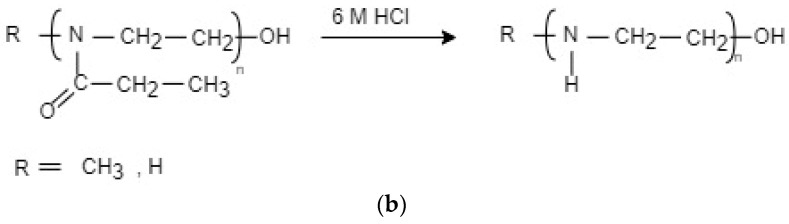
Schematics of (**a**) representation of the synthesis of LPEI via cationic ring-opening polymerization of 2-ethyl-2-oxazoline followed by acid-catalyzed hydrolysis of the resulting poly(2-ethyl-2-oxazoline), and (**b**) synthesis of LPEIs with two different starting groups (i.e., a methyl group and a proton).

LPEI derivatives were synthesized by Fernandes et al. [25] through a two-step process. Initially, poly(2-ethyl-2-oxazoline) (PEtOx, ~10,000 g/mol) was obtained via living cationic ring-opening polymerization of 2-ethyl-2-oxazoline using methyl p-toluenesulfonate as the initiator in acetonitrile at 65 °C for 60 h and, subsequently, termination with KOH in methanol. The polymer was then partially hydrolyzed using 5 M HCl at 100 °C, with samples collected at 3, 5, and 7 h to yield PEI30, PEI70, and PEI96 copolymers, respectively. Hydrolysis was monitored by ^1^H NMR, where the backbone peak shifted from 3.45 to 2.90 ppm. PEI70 showed optimal gene delivery performance with strong nucleic acid binding and low cytotoxicity, as shown in Figure 1a. In a similar way, Jäger et al. [26] prepared LPEI by fully hydrolyzing PEtOx (1–50 kDa), synthesized using methyltosylate or p-toluenesulfonic acid in acetonitrile at 140 °C. Hydrolysis with 6 M HCl at 130 °C for 1 h under microwave heating achieved >99% conversion, validated by NMR and mass spectrometry. The resulting LPEIs were highly pure, with tunable-end-group functionalities suitable for pharmaceutical applications, as shown in Figure 1b.

### 2.2. Polymerization of Branched PEI

Branched polyethylenimine (BPEI) is a highly cationic polymer composed of a complex structure of primary, secondary, and tertiary amine groups. It is primarily obtained through the ring-opening polymerization of ethyleneimine in acidic environments, where nitrogen protonation of the aziridine ring facilitates nucleophilic attack on one of the carbon atoms, initiating polymer chain growth. The aziridine monomers and terminal rings, being more basic than the polymer backbone, preferentially bind H^+^ ions to form highly reactive aziridinium rings that are prone to nucleophilic attack [27]. This process, often aided by strong acids such as HCl or H_2_SO_4_, enables the creation of dense cationic networks. BPEI’s high charge density and broad hydrogen bonding capacity make it useful in gene delivery, antimicrobial coatings, flocculants, and adhesives. Its appeal in biomedical applications is further enhanced by its broad molecular weight range and the simplicity of its industrial production, which is frequently achieved via intermolecular dehydration of 2-aminoethanol [12].

Brodie et al. [28] developed a direct catalytic method for synthesizing BPEI through hydrogen-borrowing polymerization of ethanolamine. The reaction was catalyzed by a manganese pincer complex in toluene at 150 °C under mild base conditions, enabling sequential dehydrogenation, imine coupling, and hydrogenation steps. The resulting polymer exhibited a branched architecture, with a number-average molecular weight (M_n_) of approximately 38.7 kDa and a dispersity of 1.2, obtained in an 81% yield. This aziridine-free process offers a safer, scalable, and ecofriendly route to BPEI for biomedical and industrial applications.

**Figure 2:**
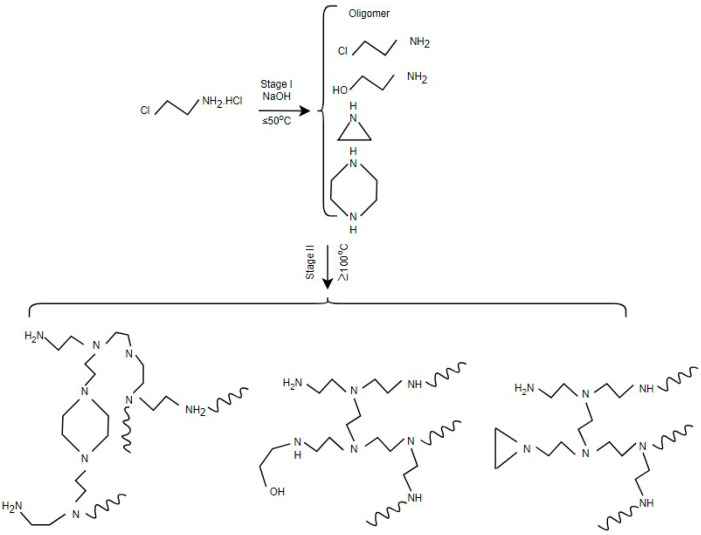
Schematic of the one-pot two-stage synthesis of branched PEI from 2-chloroethylamine hydrochloride.

Apart from the previously mentioned challenges, another problem is the difficulty in controlling molecular weight and level of branching since the tendency of the polymerization reaction is to result in a wide distribution of chain lengths. Zhang et al. [29] introduced a new one-pot, two-stage process, as shown in Figure 2, that is capable of overcoming these obstacles, providing a safer and more economic option. In stage-I, 2-chloroethylamine is dehydrochlorinated intramolecularly in an aqueous NaOH solution at 25 °C for 24 h to produce aziridine. This was generated directly within the reaction mixture with a yield of approximately 77.5%, along with trace byproducts such as PEI oligomers, piperazine, and ethanolamine. In stage II, the reaction mixture is heated at 100 °C for another 24 h to induce ring-opening polymerization of aziridine. Along the way, existing PEI oligomers serve as chain extenders and branching agents. The resulting PEI is of a controlled molecular weight with about 4% impurities in the form of hydroxyethyl end groups and piperazine rings without compromising its thermal stability.

**Figure 3:**
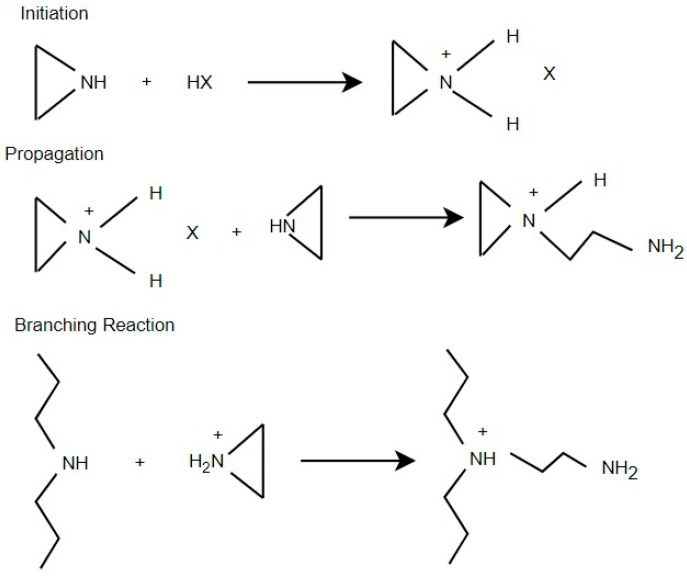
Acid-catalyzed ring-opening polymerization mechanism of aziridine, illustrating the initiation, propagation, and branching steps involved in the formation of branched polyethyleneimine (BPEI).

An alternative synthesis method for BPEI, shown in Figure 3, was reviewed by Gleede et al. [30]. The reaction is initiated by protonation of aziridine under acidic conditions (e.g., hydrochloric acid), followed by inter- and intramolecular nucleophilic attack of the protonated monomer to propagate and introduce branching. Polymerization typically occurs in aqueous or polar solvents at temperatures ranging from room temperature to 100 °C, with reaction times varying from hours to several days depending on the monomer-to-catalyst ratio and temperature. Molecular weights ranging from 5 to 30 kDa with highly branched architectures can be achieved. Characterization using NMR and MALDI-TOF MS confirm the complex, multimodal molecular weight distribution and nonlinear topology of the resulting polymers. Their review also discusses improved safety protocols and alternatives to mitigate the hazards associated with aziridine handling, making it a valuable modern reference for acid-catalyzed BPEI synthesis.

## 3. Processing and Structure–Property of PEI Carriers

### 3.1. Nanoparticles

Jin et al. [18] discussed the processing of PEI-based copolymers into nanoparticles to optimize their size, stability, and siRNA encapsulation efficiency. Nanoprecipitation was employed as the primary technique for the self-assembly of core–corona nanoparticles. Specifically, PEI–PCL (800 Da–40 kDa) and PEG–PCL (5–4 kDa) were dissolved in 400 μL of THF at 2 mg mL^−1^ and 0.05 mg mL^−1^, respectively, and added dropwise into 800 μL of 10 mM HEPES buffer under stirring at 350 rpm, followed by solvent evaporation for 3 h under a fume hood to yield blank nanoparticles. The resulting nanoparticles had an average size of ~100 nm and a zeta potential ranging from +20 to +40 mV depending on the preparation method. Although the PEI–PCL–PEI/PEG–PCL system showed high siRNA encapsulation and improved cellular uptake, while revealing a significant limitation in gene silencing performance due to structural restrictions on siRNA release, the tightly packed core prevented intracellular release, with only ~19.3% and ~29.1% siRNA releases at neutral pH 7.4 and 5.0, respectively, confirmed through a heparin competition assay and highlighting limited liberation from the nanoparticle core. Gene knockdown improved from ~35% to ~66% after chloroquine treatment, which disrupted the endosomal membranes. These results indicate that endosomal escape was a major barrier. Thus, while the block copolymer design enhanced nanoparticle stability and uptake, it compromised release efficiency unless aided by responsive features.

In addition to PEI–PCL copolymers, Ratanajanchai et al. [21] used an alternative method to produce PEI nanoparticles with a core–shell structure, where PEI served as the shell to encapsulate a core consisting of either polystyrene (PS), poly(methyl methacrylate) (PMMA), or poly(2-hydroxyethyl methacrylate) (pHEMA). The process involved a one-step surfactant-free emulsion polymerization that was photoinitiated by visible light using 0.02 M camphorquinone as the photoinitiator in the presence of branched PEI (M_n_ = 60,000 g/mol, 10 wt% aqueous solution). In a 50 mL reaction volume, 2 mL of monomer (styrene, MMA, or HEMA) was added to the PEI solution under nitrogen purging and irradiated for 3 h using a 300 W halogen light source placed 25 cm from the reactor at 25 °C. This resulted in PEI-immobilized core–shell nanoparticles with an average size of 127–158 nm and a surface zeta potential of +36 to +43 mV. The PEI shell provided colloidal stability and reduced cytotoxic interactions by limiting access to cationic groups. The grafting efficiency values were 84% for PEI/PHEMA, 69% for PEI/PMMA, and 51% for PEI/PS, while the number of accessible amine groups per particle was ~6.9 × 10^5^, ~1.3 × 10^6^, and ~4.6 × 10^6^, respectively. An MTT assay showed 100% cell viability after 24 h incubation at PEI concentrations up to ~35–95 µg/mL, confirming the biocompatibility of the immobilized system. These results show covalent surface immobilization of PEI can reduce cytotoxicity while maintaining cellular uptake potential, making these core–shell nanoparticles promising for gene therapy and intracellular drug delivery.

**Figure 4:**
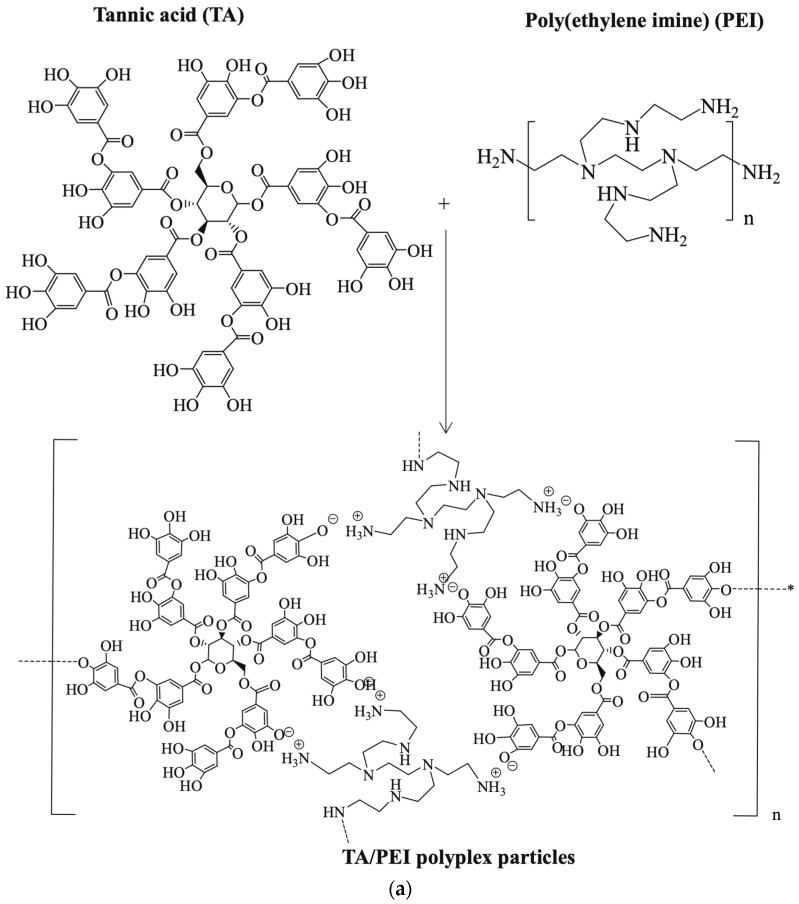

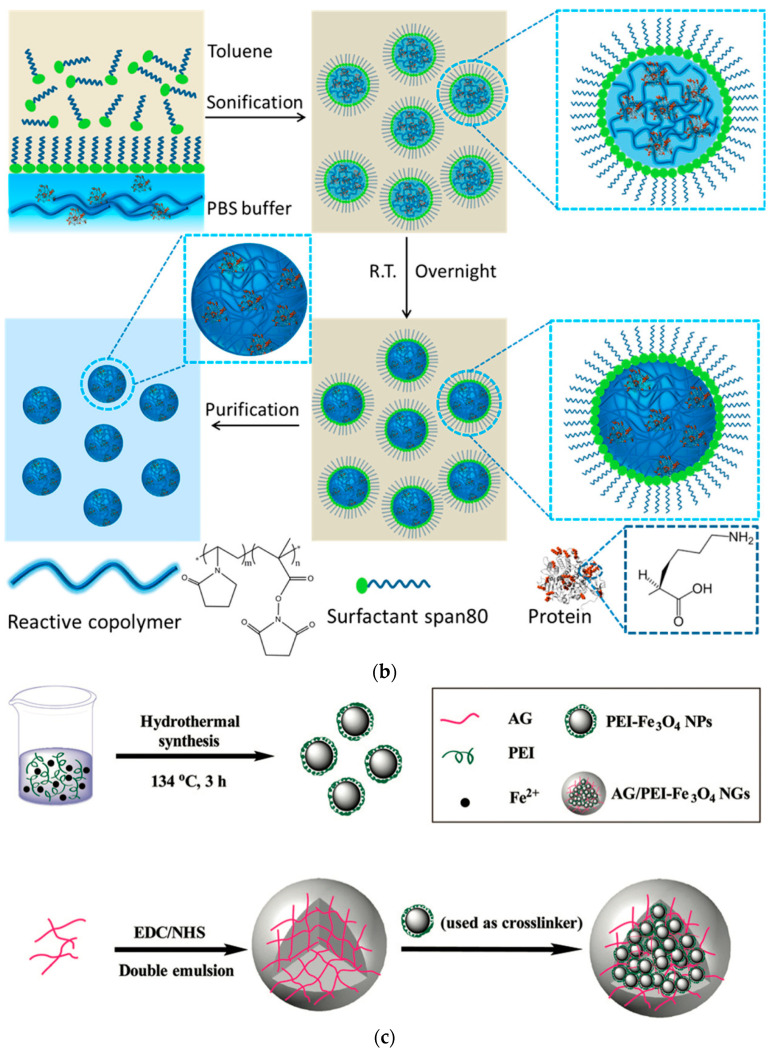
Representative synthesis strategies of PEI-based nanogels (NGs): (**a**) Formation via electrostatic complexation between PEI and tannic acid (TA). Reproduced or adapted from [32], with permission from Elsevier, 2025. (**b**) Crosslinking in a water-in-oil (W/O) emulsion using functionalized PEG. Reproduced or adapted from [33], with permission from ACS Publications, 2025. (**c**) Incorporation of PEI-stabilized nanoparticles (e.g., Au or Fe_3_O_4_ NPs) as crosslinkers into polymer matrices such as alginate (AG). Reproduced or adapted from [34], with permission from Royal Society of Chemistry, 2025. These approaches allow fine control over NG structure, size, and functional content for biomedical applications.

PEI-based nanogels (NGs) and their potential applications for gene delivery, drug delivery, and bioimaging were reviewed by Zou et al. [31] (Figure 4). PEI-based NGs were processed by crosslinking PEI with other molecules, polymers, and reactive agents using three main strategies: (1) electrostatic complexation with negatively charged molecules such as tannic acid; (2) water-in-oil (W/O) emulsion methods, where aqueous PEI is crosslinked within oil droplets to form nanogels typically ranging from 10 to 100 nm; and (3) incorporation of PEI-stabilized nanoparticles as crosslinkers into pre-formed polymer networks. These approaches allow tuning of NG size and structure through control over PEI ratio, crosslinker, and reaction conditions. For example, a W/O emulsion was prepared with 10% methacrylated PEI using bis(3-aminopropyl)-PEG as a crosslinker and PBS buffer, followed by ultrasonication for 6 min to disperse the aqueous phase in the oil phase and overnight stirring for crosslinking. The resulting mixture was purified by dialysis, yielding NGs with an average diameter of about 473 nm and a narrow size distribution. In the third strategy, PEI-stabilized nanoparticles such as Fe_3_O_4_ or Au NPs were synthesized separately by hydrothermal or reduction methods, then covalently embedded into alginate-based networks via EDC coupling in a W/O/W emulsion, yielding hybrid nanogels with average sizes between 108 and 186 nm depending on composition. These methods produce nanogels with a tunable loading capacity, responsiveness, and integrity tailored for specific applications.

Bonner et al. [19] introduced a crosslinked linear polyethylenimine (xLPEI) system that integrated either non-degradable or disulfide-responsive linkages. Crosslinking of low-molecular-weight LPEI (2.5 kDa) was achieved using EDC/NHS chemistry in the presence of either succinic acid for non-degradable crosslinking or dithiodipropionic acid for degradable disulfide linkages in a methanol/dimethyl sulfoxide solvent system. The xLPEI had molecular weights ranging from 30,000 to 50,000 g/mol, particle sizes of ~350–500 nm, and zeta potentials between +20 and +40 mV, depending on the degree of crosslinking. Following crosslinking, the xLPEI was then complexed with plasmid DNA (pDNA) through electrostatic interaction in phosphate-buffered saline to form polyplexes designed for efficient cellular uptake and intracellular gene delivery. At 4% crosslinking, transfection efficiency reached ~40% in KB cancer cells, with no detectable toxicity compared to conventional 25 kDa branched PEI. Notably, free xLPEI chains—not disulfide-degradable—were found to play a key role in endosomal escape, achieving >75% cytosolic release without membrane damage. This challenges the assumption that disulfide reduction is essential for unpacking, highlighting instead the importance of free polymer availability and structural mobility in enabling efficient delivery. These findings suggest that polymer architecture and intermolecular behavior may be as important as chemical degradability in the design of safer, more effective non-viral vectors.

**Figure 5:**
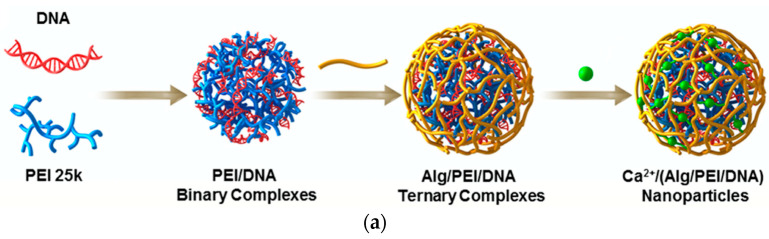

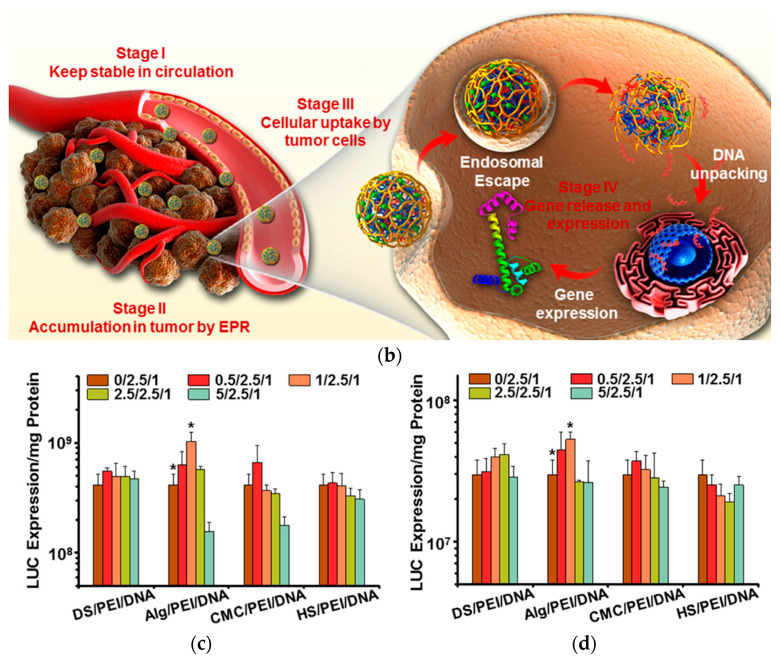
Schematic representation and transfection performance of polysaccharide-modified PEI/DNA delivery systems. (**a**) Formation process of Ca^2+^-crosslinked Alg/PEI/DNA nanoparticles, progressing from PEI/DNA binary complexes to ternary complexes with alginate, and final stabilization via Ca^2+^ coordination. (**b**) Proposed intracellular delivery mechanism of the nanoparticles, involving systemic circulation, tumor accumulation via the EPR effect, cellular uptake, endosomal escape, DNA unpacking, and subsequent gene expression. (**c**) Luciferase gene expression in HeLa cells transfected with various polysaccharide/PEI/DNA complexes at different N/P ratios. (**d**) Corresponding transfection results in HepG2 cells. Data represent mean ± SD (n = 3); * *p* < 0.05 indicates significant difference. Reproduced or adapted from [16], with permission from Elsevier, 2025.

Zhang et al. [16] prepared PEI/DNA complexes by mixing PEI and DNA solutions in a 2.5:1 weight ratio for gene therapy. Polysaccharide solutions (e.g., alginate, heparan sulfate, dextran sulfate, or carboxymethyl cellulose) were added at polysaccharide/PEI/DNA weight ratios of 0.5/2.5/1, 1/2.5/1, 2.5/2.5/1, and 5/2.5/1 followed by 20 min of incubation to prepare various polysaccharide/PEI/DNA complexes. Calcium was then added and incubated for 20 min at room temperature to prepare ionic-crosslinked nanoparticles. Transfection efficiencies were evaluated using HeLa and HepG2 cells with luciferase-encoded plasmid DNA. Among all polysaccharides tested, alginate-based PEI/DNA complexes at a ratio of 1/2.5/1 showed the highest luciferase expression in both cell lines, indicating their enhanced gene delivery capability (Figure 5). The alginate layer acted as an optimal shield. The layered structure of the Ca^2+^/(alginate/PEI/DNA) nanoparticles offered pH-responsive release behavior and enhanced biodistribution. The outer alginate shell, ionically crosslinked with calcium ions, formed a semi-permeable network that exhibited pH-sensitive and ion-strength-dependent swelling, enabling selective DNA release in acidic tumor microenvironments. Gel retardation tests confirmed that DNA was tightly retained at pH 7.4 with no visible migration, while a clear release was observed at pH 5.5, indicating pH-responsive disassembly of the nanoparticle complex. Quantitatively, less than 10% of DNA was released at pH 7.4 over 24 h, whereas over 60% of DNA was released under acidic conditions. Additionally, calcium crosslinking significantly reduced the initial burst release, where only ~13.5% of DNA was released within the first 12 h from Ca^2+^-crosslinked particles versus ~45% from uncrosslinked controls, highlighting the role of Ca^2+^ in decreasing porosity and improving in vivo structural stability. Biodistribution assays further demonstrated tumor accumulation of ~23.4% of the injected dose at 12 h post-injection, which was attributed to enhanced payload protection and release kinetics tuned for acidic tumor microenvironments. Collectively, these features demonstrate how structural layering and ionic crosslinking together optimize pharmacokinetics, biodistribution, and release behavior.

Wu et al. [35] employed low-molecular-weight PEI (1.2 kDa) to create PEI/protein complexes via electrostatic interaction. Proteins such as GFP, β-galactosidase, DNase I, BSA, and HAND2 were mixed with PEI at various N/P ratios (2:1 to 16:1) to optimize complex formation and intracellular delivery. The components were gently mixed in phosphate-buffered saline (PBS) and allowed to self-assemble for 15 min at 4 °C, with no chemical modification required—preserving protein structure and function. These nanocomplexes exhibited hydrodynamic diameters ranging from 244 to 359 nm, with optimal condensation at N/P = 8. Zeta potential ranged from +15.4 to +45.3 mV, promoting membrane interaction and cellular uptake. TEM confirmed spherical morphology and uniform dispersion. Protein integrity was preserved, with β-galactosidase maintaining catalytic activity inside HDFs and hBMSCs, as confirmed by X-gal staining. The encapsulation efficiency reached up to 98.3%, and protein release persisted for up to 96 h without premature degradation. These findings underscore the structural competence of PEI 1.2 kDa for protein delivery—achieving high uptake, stability, and bioactivity with minimal cytotoxicity below 1 µg/mL.

Numerous reports detailed the capabilities of nanoparticle-forming polymers in delivering nucleic acid therapeutics with high efficiency and low toxicity [36]. PEI is considered one of the most extensively investigated cationic polymers due to its ability to interact with the negatively charged backbone of nucleic acids and form nanosized particles. PEI’s effectiveness in gene transport is directly influenced by its molecular structure, which depends on its branching, molecular weight, and amine concentration. While these characteristics contribute to PEI’s effectiveness in gene transport, they also introduce toxicity concerns. To improve the practical usability of modified PEI carriers in gene therapy, ongoing research seeks to preserve their beneficial traits while reducing their adverse effects.

The ability of PEI to interact with various ions and molecules, particularly transition metal ions, is significantly influenced by its high density of amine groups. These amine groups act as electron donors to enable PEI to effectively coordinate with metal ions, such as Au^3+^, Pd^2+^, and PtCl_6_^2−^, forming stable metal colloids and nanoparticles in aqueous solutions. For instance, Bronstein et al. [37] demonstrated that the interaction of PEO-*b*-PEI with H_2_PtCl_6_ at a molar NH:Pt ratio of 4:1 yielded narrowly distributed micelles with hydrodynamic diameters of ~154.3 nm, which could be reduced to ~110.0 nm upon hydrazine treatment. Similarly, AuCl_3_ interactions at a NH:Au ratio of 12:1 led to micelles of ~62.5 nm in diameter, with gold nanoparticle cores averaging ~29.7 nm in size. These results show that a lower metal loading (e.g., NH:Au = 24:1) yields smaller particles (~6.0 nm), while a higher loading can result in micelle destabilization and aggregation.

This stabilizing role of PEI is not limited to self-assembled systems. Zhou et al. [38] demonstrated that PEI-modified silica supports enabled the dispersion of Pd(0) nanoparticles with diameters in the range of 2.5–4 nm, improving hydrogenation activity due to PEI’s chelation of Pd^2+^ and steric prevention of aggregation. Such micelle-assisted reductions and complexation not only stabilize the nanoparticles but also provide size control through the polymer architecture and reactant ratios. The ability to fine-tune size and shape via PEI’s coordination with metal ions underpins its widespread use in catalysis, nanomaterial synthesis, and gene transport applications [37,39].

Park et al. [17] characterized HA-shielded PEI/pDNA nanogels and found that increasing the HA ratio from 1:1 to 1:10 led to a size increase from ~70 nm to ~150 nm and a surface charge shift from +22.3 mV to −13.2 mV. Confirmed via DLS, AFM, SEM, and zeta potential analysis, these structural and electrostatic changes reflect how polyanion complexation can modulate nanoparticle morphology and colloidal stability for enhanced delivery performance.

Polyethylenimine’s (PEI) structural features—such as high amine density and cationic backbone—enable it to form nanoparticles with a hydrodynamic diameter of 176.5 ± 10.2 nm, a zeta potential of +17.6 mV, and a polydispersity index of 0.281. In methotrexate (MTX) delivery, Abolmaali et al. [20] reported that these PEI-based nanonetworks achieved an encapsulation efficiency of 86.8% and drug loading of 13.2%. Under reducing conditions (10 mM DTT), they released 75% of the drug in 48 h, compared to 32% in non-reducing media. Cytotoxicity studies showed >90% cell viability at concentrations up to 100 µg/mL, and therapeutic testing indicated an IC_50_ of 2.8 µg/mL for MTX-loaded nanoparticles versus 5.1 µg/mL for free MTX. These quantitative outcomes directly reflect how PEI’s structure governs its drug delivery functionality.

One major drawback of PEI-based nanoparticles is derived from their overall cationic charge, which results in non-specific interactions with serum proteins, as well as negatively charged components from non-targeted cells. This cytotoxicity arises from the disruption of cellular organelles such as mitochondria and lysosomes due to strong electrostatic interactions between PEI and intracellular membranes. In addition, the non-biodegradable property of PEI prevents rapid elimination and therefore contributes to some level of cellular toxicity [40]. Thus, great attention has been raised to designing PEI nanoparticles that offer excellent biodegradability with the ability to prevent non-specific target interactions.

### 3.2. Coatings

Li et al. [41] utilized PEI as a surface coating for a wide range of nanoparticles, including magnetic Fe_3_O_4_, gold, gadolinium- and manganese-based MRI agents, silica nanostructures, upconversion nanoparticles, and carbon-based materials. In this context, PEI acts as a size-controlling agent and colloidal stabilizer, reducing nanoparticle aggregation and improving monodispersity. For example, Fe_3_O_4_ nanoparticles (~10 nm) were coated with 1.2 kDa PEI by stirring at room temperature for 12 h, while gold nanoparticles were formed by reducing HAuCl_4_ with PEI at 90 °C for 30 min. Coated Fe_3_O_4_ particles exhibited size reductions from ~31.1 nm to 11.5–21.8 nm and r_2_ relaxivity up to 461.3 mM^−1^·s^−1^. The high density of PEI’s surface amines allowed conjugation with Gd^3+^, siRNA, folic acid, and fluorescent dyes, enabling PEGylation and acetylation to modulate surface charge and circulation. These modifications extended blood circulation half-life, while PEI–Au nanoparticles reached ~11.2 h, enhancing their use in CT and multimodal imaging. Together, these features demonstrate PEI’s versatility as a multifunctional scaffold for drug loading, targeting, and diagnostic applications in cancer nanomedicine.

Sun et al. [42] coated carbon nano-onion clusters (CNOCs) with PEGylated PEI for cancer theranostics. The CNOCs were synthesized by sonicating candle soot in a sulfuric acid/nitric acid mixture at 50 °C for 4 h, followed by purification and water dispersion (hydrodynamic size ≈ 90 nm). Low-molecular-weight PEI (600 Da) was conjugated to CNOCs via carbodiimide coupling to carboxyl groups, followed by PEG grafting using NHS-activated PEG (5 kDa), resulting in CNOCs–PEI–PEG with a hydrodynamic size of ~145 nm and zeta potential of +25.9 mV. The coating shifted the zeta potential from −54.3 ± 9.1 mV to +25.9 ± 7.5 mV and increased the size from ~90 ± 31 nm to ~145 ± 55 nm, confirming successful surface modification. These coated CNOCs exhibited a photothermal conversion efficiency of 56.5% and an ultrahigh cellular uptake of ~21.3 pg/cell, with visible lysosomal aggregation enhancing intracellular heat localization—an essential feature for effective photothermal therapy. Together, structural design and surface engineering resulted in stable, biocompatible nanofilms with excellent photothermal performance and enhanced cellular uptake.

Sun et al. [43] used linear PEI as a reducing and protecting agent to prepare gold nanoparticles in a single-step thermal synthesis. The synthesis involved heating aqueous solutions containing HAuCl_4_ (0.0243 mol/L) and linear PEI (M_w_ = 423) at 60 °C for several minutes, using varying initial PEI-to-gold molar ratios (2:1 to 9.5:1). In addition to the reduction of HAuCl_4_ to metallic gold, linear PEI was used to stabilize and shape the gold nanoparticles as a coating polymer. Monodispersed spherical particles (~25 nm in diameter) were obtained at ratios between 5:1 and 6.5:1, while quasi-one-dimensional aggregates formed at a lower LPEI-to-gold ratio of 2:1 and at a higher LPEI-to-gold ratio of 9.5:1. The results confirm the versatility of PEI as a surface-active polymer with dual functions, including the ability to catalyze nanoparticle production and to provide electrosteric stabilization of these nanoparticles.

In a study by Gorejová et al. [44], a thin PEI layer was applied via dip-coating onto sintered iron pellets to investigate their surface interactions and degradation effects. The PEI was branched, with a molecular weight of 60–100 kDa, applied from a 5 wt% solution for 90 min and dried at 37 °C for 12 h. AFM and SEM showed that PEI formed longitudinal polymer wave structures concentrated in valleys, while high protrusions remained partially uncoated, indicating topographically selective coverage. AFM also confirmed ~50 nm iron subgrain sizes in uncoated samples. DFT modeling showed that PEI monomers bind to Fe(110) surfaces via their terminal –NH_2_ group, with an Fe–N bond length of 2.11 Å, an adsorption energy of −1.38 eV, and an Fe–N–C angle of 122.9°, confirming stable chemisorption. Electrochemical impedance spectroscopy further confirmed polymer adsorption, revealing the formation of a second semicircle after 10 min, indicative of surface film formation. Compared to the uncoated Fe, PEI-coated samples exhibited lower charge transfer resistance (Rct = 8.25 Ω vs. 13.9 Ω) and higher double-layer capacitance (Qdl = 2.82 µF vs. 0.70 µF), demonstrating increased surface reactivity and corrosion potential due to the PEI layer.

### 3.3. Fibers

**Figure 6:**
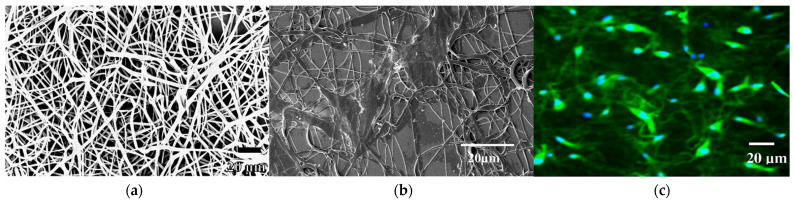
Electrospun PEI fibers for cell cultures. (**a**) SEM image of electrospun linear PEI scaffold, showing an interconnected fibrous network. (**b**) SEM images of electrospun linear PEI scaffolds with adhered NHF cells following 5 days of culture. (**c**) Fluorescence microscopy image using DAPI (blue) and anti-GRP78 conjugated with Alexa Fluor 488 (green), showing NHF cells on linear PEI scaffolds. Reproduced or adapted from [45], with permission from Elsevier, 2025.

Khanam et al. [45] fabricated fibers directly from linear PEI, which is uncommon due to its solubility and cytotoxicity. To overcome these issues, 400 mg of linear PEI was first dissolved in 2 mL methanol and reacted with 80 mg succinic anhydride to partially neutralize its primary amines and reduce overall charge density. To stabilize the structure and make the polymer insoluble in aqueous media, 0.25 mL of 1,4-butanediol diglycidyl ether was added for chemical crosslinking. The resulting viscous gel (measured at 1620 cP) was electrospun using a flat-tip stainless-steel needle under a high voltage of 18 kV, with a flow rate of 0.01 mL/min and a 10 cm tip-to-collector distance. Fibers were collected on a rotating mandrel (0.7 Hz) and vacuum-dried at 45 °C overnight to remove solvents (Figure 6).

The scaffolds consisted of nonwoven PEI fibers with diameters ranging from 687 ± 71 nm to 1600 nm, pore diameters of 16.77 ± 9.16 µm, and a thickness of ~30.9 µm, forming a porous 3D structure suitable for supporting the attachment and growth of normal human fibroblasts (~20.3 µm). Structural analysis confirmed 25.2% crosslinking of amines and 3.1% carboxylate incorporation, contributing to a mechanically stable and biocompatible scaffold with reduced cytotoxicity. Over a 5-day culture period, NHF cells showed >95% viability, with a doubling time of 74 h, comparable to the control. SEM, fluorescence, and DIC imaging confirmed that cells adhered to, spread within, and penetrated the fibrous mesh, demonstrating robust 3D integration and confirming the scaffold’s suitability for tissue engineering applications.

**Figure 7:**
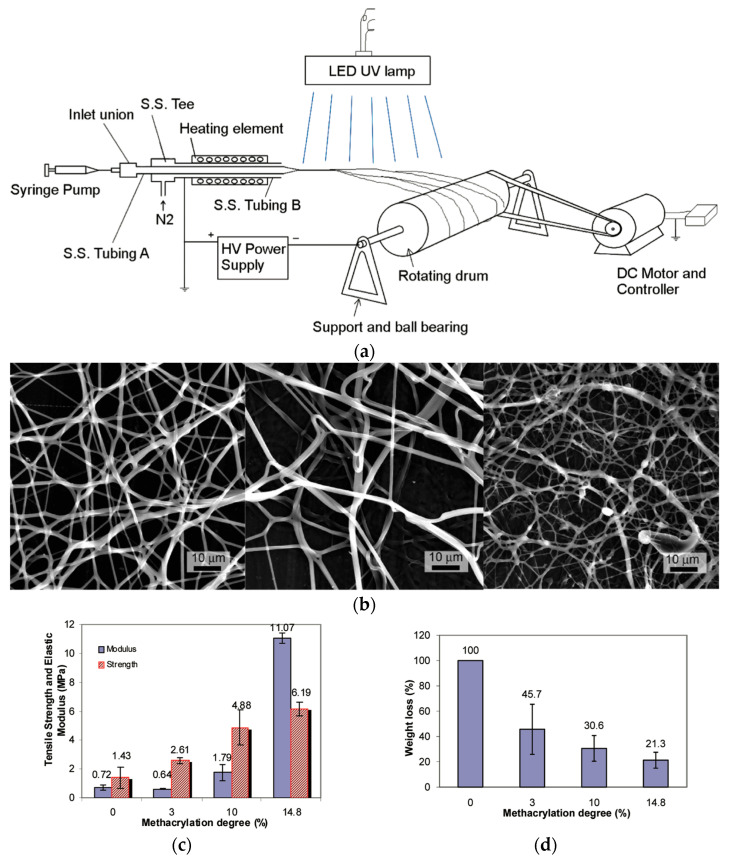
Structure–property relationships of PEI fibers. (**a**) Simplified schematic of the reactive electrospinning setup. (**b**) SEM images of the morphology of linear PEI fibers combined with 2% PVP and crosslinked under varying conditions. From left to right: 14.8% methacrylation crosslinked at 140 mW/cm^2^, 14.8% methacrylation crosslinked at 191 mW/cm^2^, and 59.2% methacrylation crosslinked at 140 mW/cm^2^. Scale bar: 10 μm. (**c**) Average tensile strength and average elastic modulus of methacrylated linear PEI fibers with increasing methacrylation degree, measured after UV curing. (**d**) Average weight loss of crosslinked linear PEI fibers after 48 h ethanol immersion, showing reduced solubility with higher methacrylation degrees. Reproduced or adapted from [46], with permission from ACS Publications, 2025.

Xu et al. [46] processed LPEI into crosslinked fibers using a reactive photo-electrospinning technique. LPEI (M_w_: 250,000 g/mol) was chemically modified with glycidyl methacrylate in chloroform to introduce photo-crosslinkable methacrylate groups. The typical functionalization level used was a 14.8% methacrylation degree. The product was purified by precipitation in acetone, then dried and stored at 4 °C in foil-wrapped containers to prevent premature crosslinking. To enable fiber formation, 2% polyvinylpyrrolidone (PVP, M_w_ ≈ 1,300,000 g/mol) was added to the methacrylated LPEI solution to enhance chain entanglement, along with a 1% photoinitiator, phenyl-bis(2,4,6-trimethylbenzoyl)–phosphine oxide (PO). The optimized electrospinning solution consisted of 10% methacrylated PEI, 2% PVP, and 1% PO in ethanol. Electrospinning was performed using a heated spinneret at 40–43 °C, with a flow rate of 0.3 mL/h, and fibers were collected on a rotating drum (800 rpm) placed 14 cm from the tip. In situ UV curing was applied with a UV lamp at 140–191 mW/cm^2^ placed 16 cm from the jet during spinning (Figure 7). The resulting crosslinked PEI fibers had average diameters ranging from 419 ± 256 nm to over 2 µm, depending on the UV intensity and methacrylation degree. Crosslinked fibers exhibited 78.7% weight retention after 48 h in ethanol, confirming solvent resistance. DSC analysis showed no melting point, indicating restricted chain mobility due to dense crosslinking. Mechanical strength and elastic modulus increased with methacrylation level. Water contact angles increased from 57.0° to 86.8°, confirming enhanced hydrophobicity. The in situ photo-crosslinking avoided the need for post-treatment, minimized residual toxicity, and maintained a fibrous morphology in aqueous and ethanol media, making this method highly suitable for biomedical applications.

### 3.4. Hydrogels

A novel technique for creating LPEI-based hydrogels was demonstrated by Schubert et al. [47], with the goal to facilitate effective DNA binding and release. The process started with acidic hydrolysis of poly(2-ethyl-2-oxazoline) (PEtOx, 50,000 g/mol) in 6 M HCl under microwave heating at 130 °C for 1 h to obtain LPEI, followed by functionalization of LPEI with butenyl groups through an amidation reaction using *N*-succinimidyl-4-pentenate to form a copolymer of poly(2-(3-butenyl)-2-oxazoline-*co*-ethylene imine) (P(ButEnOx-*co*-EI)). This copolymer enabled crosslinking through thiol–ene photopolymerization under UV light (365 nm for 24 h with 3,6-dioxaoctane-1,8-dithiol as crosslinker and 2,2-dimethoxy-2-phenylacetophenone as photoinitiator) without lowering the quantity of functional amine groups required for genetic material interaction. By varying the degree of crosslinking, the resulting hydrogels’ swelling behavior could be controlled, with values ranging from ~74% at 50% PEI to 23% at 5% PEI, correlating with reduced pore sizes from ~60–70 μm to ~20–30 μm. The hydrogels retained high PEI functionality, allowing efficient DNA binding (~70% fluorescence quenching at high PEI content), as demonstrated by ethidium bromide assays, while release was achieved via heparin treatment, with copolymers releasing up to 90% of DNA rapidly at room temperature and hydrogels requiring combined heparin and heat (~90 °C) to release ~50%. These findings highlight that network architecture, particularly crosslinking density and PEI content, governs water uptake and electrostatic interactions essential for gene delivery, biosensing, and nucleic acid purification applications.

To operate as local medication delivery implants for sockets that have had teeth extracted, Chang et al. [48] created a composite hydrogel system made of PEI and poly(vinyl pyrrolidone) (PVP) crosslinked with different glutaraldehyde concentrations. The hydrogels were formed by mixing a 0.75% *w*/*v* PEI solution with PVP K90, incorporating lidocaine HCl, and then crosslinking the mixture with various concentrations of glutaraldehyde, followed by drying. Notably, the crosslinking density had little effect on the drug release rate but impacted the swelling ratio as it decreased from ~1150% to ~450% as glutaraldehyde concentration increased from 0% to 0.5% due to a tighter mesh structure. However, lidocaine release remained largely unaffected, indicating that diffusion was governed more by hydration dynamics than network density. Cumulative lidocaine release reached ~97% within 3 h, with rate constants of ~38–40%/h across all crosslinking levels. This decoupling between mesh density and drug diffusion enables independent tuning of hydration and release behavior. MTT assays on gingival fibroblasts showed ~76% cell viability for hydrogels crosslinked with 0.5% glutaraldehyde, compared to 90% for clinical-grade Spongostan^®^, indicating acceptable but slightly reduced biocompatibility. In vivo testing in beagle dogs confirmed that PEI–PVP hydrogels maintained their structure in the socket and accelerated wound healing comparably to Spongostan^®^, supporting their potential as resorbable drug-delivering wound dressings.

Avais and Chattopadhyay [49] used a bifunctional azetidinium-based crosslinker to crosslink branched PEI under aqueous conditions and create new waterborne pH-responsive hydrogels. In the one-pot synthesis, an aqueous solution of PEI (1 g in 10 mL water) was mixed with a defined amount of the crosslinker (10%, 20%, or 30% *w*/*w* relative to PEI amines) and heated at 80 °C for 3 h, enabling a network formation through azetidinium–amine reactions without the need for catalysts or additives. PEI’s high density of protonatable amine groups and pKa around physiological pH (~7.2) were utilized in the novel design to allow the hydrogels to show selective maximal swelling at pH 7.4. Compared to conventional pH-responsive hydrogels, which usually exhibit progressive swelling responses without a dramatic peak close to physiological pH, this behavior was noticeably different. According to this study, PEI-10 hydrogels had the largest water uptake (1300% swelling ratio), and the swelling capacity declined as the crosslinking density increased (850% and 650% for PEI-20 and PEI-30, respectively). Additionally, their pH-responsiveness resulted in the controlled and selective release of a model drug (Rhodamine B), with the highest release occurring at pH 7.4 (>60% cumulative release in 24 h). The hydrogels demonstrated swelling-controlled drug delivery with near-zero-order release kinetics from 2 to 12 h (R^2^ ≈ 0.94). This study showed how careful control of PEI structure and crosslinking could produce intelligent hydrogels that can target physiological conditions for use in biomedical applications, especially drug delivery.

### 3.5. Films

Wu et al. [50] created a new technique for fast-forming pyrogallol (PG) PEI nanofilms at the gas/liquid interface. The nanofilms were formed by floating polypropylene microfiltration membranes (average pore size: 0.22 µm, 75% porosity) on a PG/PEI aqueous solution (1 mg/mL PG, 2 mg/mL PEI in 200 mM Tris buffer, pH 8.5) under ambient conditions for 10 min. PG was first dissolved in water and PEI was added at a defined mass ratio, forming a uniform film at the air–water interface within 10 min. Film thickness increased nearly linearly with reaction time, from 17 nm (5 min) to ~50 nm (10 min), and up to 80 nm at 20 min. Their technique produced uniform and defect-free nanofilms that were transferred onto support membranes by a fishing method (lifting through the interface) and subsequently crosslinked with 1 wt% glutaraldehyde vapor or solution to enhance solvent resistance. Water contact angle (WCA) measurements confirmed enhanced wettability, dropping from 142° (uncoated) to ~75° post-coating. The surface charge of the nanofilms could be tuned between positive and negative values by varying the PG/PEI mass ratios, with the isoelectric point ranging from 5.6 to 7.0 depending on the ratio. This charge modulation enabled selective salt rejection, making them ideal for nanofiltration. When composited onto microporous polypropylene membranes, the nanofilms demonstrated outstanding water distillation performance with a 94% salt rejection rate using MgCl_2_ and a high-water flux of 17.5 L m^−2^ h^−1^ bar^−1^ at 0.6 MPa. These values remained stable after 120 h of continuous filtration, indicating excellent mechanical and operational durability. MTT assays showed over 85% cell viability in 3T3 fibroblasts, confirming low cytotoxicity. Furthermore, these membranes retained their chemical integrity in organic solvent nanofiltration, achieving 95.2% rejection for methyl blue and solvent permeance up to 1.40 L m^−2^ h^−1^ bar^−1^ for acetone. This method also demonstrated high atom economy, as the deposition solution was reusable for at least 12 fabrication cycles without compromising separation efficiency.

**Figure 8:**
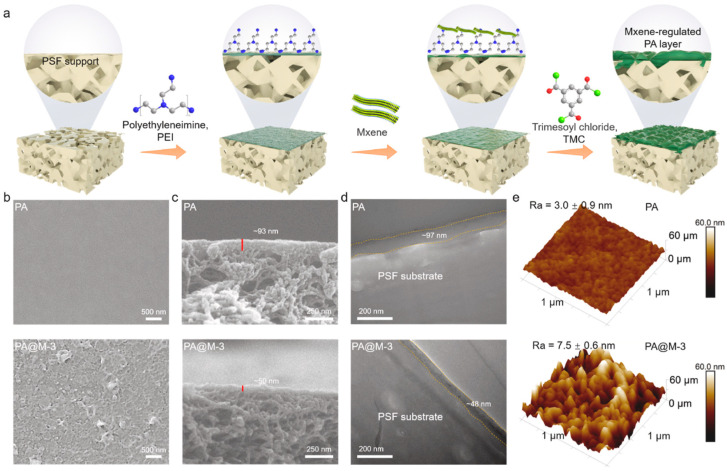
Structural and surface characterization of polyamide (PA) and MXene-regulated PA@M–3 membranes. (**a**) Schematic of membrane fabrication, including PEI and MXene modification. (**b**) Top surface SEM images, (**c**) cross-sectional SEM images, (**d**) cross-sectional TEM images, and (**e**) 3D AFM images. Reproduced or adapted from [51], with permission from Elsevier, 2025.

Wen et al. [51] developed a PEI-based nanofiltration membrane (Figure 8) via MXene-regulated interfacial polymerization (MRIP), which enabled precise control over nanofilm structure by spatially confining PEI and trimesoyl chloride (TMC) diffusion at the water–oil interface. The membrane was fabricated by sequentially applying 0.6 wt% PEI and MXene/n-hexane dispersion onto a polysulfone substrate, followed by interfacial polymerization with 0.1 wt% TMC. The membrane was thermally cured at 65 °C for 8 min to form the PA@M structure. Compared to conventional polyamide films (~93–97 nm), this method reduced thickness by 35–50%, yielding films of 48–64 nm, and increased pore size from ~0.31 nm to 0.42–0.48 nm. Surface roughness increased (Ra from 3.0 ± 0.9 nm to 7.5 ± 0.6 nm), while the water contact angle dropped from 62° to 39–49°, confirming enhanced hydrophilicity. Chemical analysis revealed a higher amine content (N–H/NH_4_^+^ = 23.8%) and decreased crosslinking (N–C=O from 85.6% to 70.2%), suggesting increased free volume and permeability. The isoelectric point also shifted from 6.15 to ~7.5–8.0, reflecting an enhanced surface charge due to greater protonation of PEI amines. Functionally, the modified membrane achieved a >300% increase in water permeance and exhibited outstanding ion selectivity, with >98% rejection for Mg^2+^ and <20% for Li^+^. In a two-stage nanofiltration process simulating salt lake brines, the Mg^2+^/Li^+^ concentration ratio was reduced from 40 to 0.45, demonstrating the membrane’s strong potential for lithium extraction. PEI-based nanofilms were thus structurally tuned to simultaneously improve permeability, selectivity, and surface properties for advanced membrane applications.

A comparative summary of various PEI formulations shown in Table 2, organized by their molecular weight (MW), transfection efficiency, and cell viability, thereby highlighting critical structure–property trade-offs, reporting transfection efficiency and cell viability, to underscore that molecular architecture—not just molecular weight—is a key determinant of therapeutic utility in PEI-based systems. Structural modifications such as PEGylation, crosslinking, and nanoparticle encapsulation have been shown to improve the safety profile of PEI while retaining or even enhancing transfection performance.

## 4. Biomedical Applications of Polyethylenimine Carriers

### 4.1. Applications

#### 4.1.1. Drug Delivery

As a cationic polymer, PEI can effectively coat or conjugate drug agents through multiple methods [14]. Biological agents like folic acid [23,52], hyaluronic acid [17,53], lactic acid [54], transactivating proteins [24], and antibodies [55] can be modified to target specific cancer cells. For example, PEI can be modified for cell marking by fluorescent labeling molecules like fluorescein isothiocyanate [22], and its biocompatibility can be enhanced by biocompatible agents like PEG and oligosaccharides [21,56]. The abundant amino groups on its surface and the internal cavity of hyperbranched PEI make it easy to create a nanoplatform that can efficiently stabilize or entrap metal ions or small biological molecules, such as DNA and siRNA. PEI’s inexpensive cost and distinct physicochemical characteristics encourage its widespread use in biomedicine. The abandonment of amino groups in PEI results in a certain degree of cytotoxicity; cationic PEI enters cells by adhering to negatively charged transmembrane heparan proteoglycans, which can cause cell damage through membrane destabilization [57].

Lactobionic acid (LA)-modified magnetite nanoparticles were created by Selim et al. [58] to allow for targeted administration to hepatocytes for liver-specific applications. To enhance cellular absorption through liver cell galactose-recognizing receptors, LA was covalently grafted onto the nanoparticle surface. The nanoparticles had a core size of ~10 nm and a hydrodynamic diameter of ~26 nm. MRI imaging verified that LA-modified nanoparticles exhibited preferential accumulation in the liver after intravenous administration in rabbits, as evidenced by contrast enhancement in liver MR images within 3 min of injection (3 mL at 4.2 mg/mL), and much higher uptake (~665 pg/cell) over 5 days, with a maximum uptake of 743 pg/cell on day 2, compared to 340 pg/cell for unmodified particles and 186 pg/cell for maltotrionic acid-coated particles. This technology demonstrated good biocompatibility and had potential for therapeutic administration or liver-targeted diagnostics.

Zhou et al. [22] used fluorescein isothiocyanate (FI) as a fluorescent tracking moiety in their multifunctional PEI-based nanocarriers for targeted anticancer drug delivery. This method enabled real-time tracking of nanoparticle dispersion and cellular uptake. The nanoplatform, which was made up of PEI that was successively modified with ~24 PEG chains, ~5.8 FA ligands, and ~4.5 FI molecules per PEI, was created to minimize cytotoxicity through acetylation (surface potential reduced to ~1.33 mV). Each PEI molecule encapsulated ~6.9 doxorubicin (DOX) molecules (3.58 wt%). The carrier displayed a pH-responsive drug release profile, releasing 51.0% of DOX at pH 5.0 and 31.5% at pH 7.4 within 50 h. By offering a fluorescent signal for observing nanoparticle behavior both in vitro and in vivo, FI played a vital diagnostic role that was non-therapeutic. The effectiveness of the targeting technique was confirmed by fluorescence microscopy and imaging investigations, which showed that the FI-labeled nanocarriers were efficiently internalized by tumor cells (DOX fluorescence ~2× higher than non-targeted carriers) and accumulated preferentially in tumor tissue. In vivo antitumor efficacy showed that the FA-mPEI/DOX complexes reduced tumor volume by over 80% compared to the control and increased survival to 80% at 87 days, compared to 60% (non-targeted), 40% (free DOX), and 0% (Normal Saline (NS) control). This study highlighted the usefulness of fluorescent labeling in multifunctional nanoplatforms.

To create biocompatible carriers for intracellular drug administration, Ratanajanchai et al. [21] used a visible light-triggered surfactant-free emulsion polymerization approach to create PEI-immobilized core–shell nanoparticles. This approach considerably decreased PEI’s cytotoxicity while maintaining its cationic binding capabilities by covalently grafting PEI onto polymer cores (PS, PMMA, or pHEMA). Grafting efficiency (GPEI) reached 83.8% for PEI/pHEMA, 69.2% for PEI/PMMA, and 51.1% for PEI/PS. PEI/PS exhibited the highest amine group density (~4.6 × 10^6^ groups/particle), while PEI/pHEMA had the lowest (~6.9 × 10^5^ groups/particle), correlating with core polarity. The nanoparticles were effectively internalized by Caco-2 cells, stayed stable, and exhibited no cytotoxicity up to 200 μg/mL for 24 h even though this concentration contained 35 μg/mL (pHEMA), 36 μg/mL (PMMA), and 95 μg/mL (PS) of PEI, compared to only 8 μg/mL tolerated for native PEI. The platform’s suitability for drug delivery, particularly with pHEMA and PMMA cores that are suitable for hydrophilic and all-purpose drug encapsulation, was supported by its favorable physicochemical profiles, even though no drugs were loaded in this study. These findings suggest a viable approach for developing secure PEI-based nanocarriers for future intracellular delivery applications.

**Figure 9:**
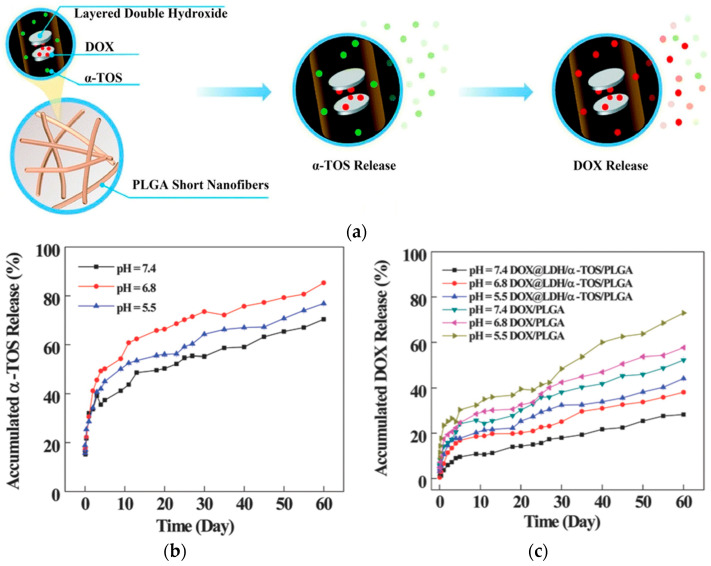
Drug delivery with short fiber systems. (**a**) Schematic of the drug release mechanism from DOX@LDH/α-TOS/PLGA short fibers, showing sequential release of α-TOS followed by DOX for MDR cancer cell treatment. (**b**) In vitro cumulative release profiles of α-TOS at different pH conditions (7.4, 6.8, and 5.5). (**c**) DOX release from DOX/PLGA and DOX@LDH/α-TOS/PLGA short fibers under the same pH conditions. Reproduced or adapted from [59,60], with permission from Royal Society of Chemistry, 2025.

Huang et al. [59] examined electrospun short fibers (SFs) as a new platform for theranostics and cancer medication delivery (Figure 9). The precise control of fiber size and shape, made possible by a variety of fabrication techniques, including homogenization, ultrasonic, and cryo-cutting, has a direct impact on therapeutic efficacy and cellular uptake. For example, homogenized doxorubicin (DOX)-loaded layered double hydroxide (LDH) nanoparticles embedded in a poly(lactic-*co*-glycolic acid) (PLGA) matrix and co-loaded with alpha-tocopheryl succinate (α-TOS) (DOX@LDH/α-TOS/PLGA) SFs had an average diameter of 830.2 nm and a mean length of 17.4 ± 7.3 μm, showing pH-dependent drug release: α-TOS released rapidly while DOX was sustained over time [59,60]. Interestingly, shorter fibers demonstrated more tumor inhibition and cellular absorption in vivo. SFs were also utilized for MRI-guided chemotherapy, with systems like DOX@PLGA-PEI-Gd/Apt fibrous rings (11.1 μm diameter, 1.69 μm fiber thickness) exhibiting high r_1_ relaxivity (4.46 mmol L^−1^ s^−1^), and for the delivery of cytokines (such as IL-2 and IL-15), it demonstrated up to 70% tumor growth inhibition through sustained local cytokine release. Dual-loaded LDH/PLGA fibers and DOX-loaded fibrous rods are two examples of drug-loaded SF systems that were highlighted as exhibiting pH-responsive, prolonged drug release and improved cytotoxicity against drug-resistant tumor cells.

In the previously mentioned study by Wu et al. [35], LMW PEI was evaluated as a carrier for the intracellular delivery of functional proteins. Using β-galactosidase (β-gal) and RNase A as model proteins, PEI–protein complexes demonstrated efficient uptake: over 80% of cells exhibited enzymatic β-gal activity, as confirmed by X-gal staining. Flow cytometry analysis further revealed that more than 85% of cells internalized the FITC-labeled protein–PEI complexes. Confocal microscopy showed strong cytosolic and perinuclear localization without significant nuclear exclusion. MTT assays confirmed minimal cytotoxicity, with cell viability remaining above 85% even after 24 h of exposure at a 10 μg/mL PEI–protein complex concentration. Moreover, the enzymatic activity of RNase A delivered by PEI led to a dose-dependent decrease in cell viability, verifying its functional release. These findings underscore the potential of low-molecular-weight PEI as a safe and effective nanocarrier for intracellular protein therapeutics.

For the co-delivery of medications and genes to tumor cells, Seo et al. [23] created a temperature-sensitive nanocarrier termed PF127-PEI-FA, which was made up of Pluronic F127, PEI, and folic acid (FA). The polymer showed a critical micelle concentration of 0.11 mg/mL, enabling stable micelle formation in aqueous conditions. The technique employed FA for receptor-mediated targeting cancer cells. The absorption was verified to be dependent on the folate receptor, as pre-treatment with 1 mM free FA reduced transfection efficiency, making it a viable option for targeted cancer treatment. The micelles allowed for controlled drug release in response to mild hyperthermia (~40 °C). Release of Nile red from the micelles increased with temperature, reaching a ~49%, ~69%, and ~77% cumulative release at 25 °C, 37 °C, and 40 °C, respectively, within 24 h. It effectively introduced plasmid DNA (5 µg of pEGFP-C1) and Nile red (0.5 ng) to HeLa cells, demonstrating robust absorption and gene expression. The technology showed minimal cytotoxicity as PF127-PEI-FA was nontoxic even at 500 µg/mL, unlike free PEI, which became cytotoxic above 10 µg/mL.

#### 4.1.2. Gene Therapy

PEI was introduced in 1995 as a cationic polymer to transport nucleic acids [61]. Since then, PEI and its derivatives have been widely utilized as gene carriers, and a few clinical applications were reported [10]. Yang et al. [52] used PEI to modify oleic acid (PEI-OA), then were non-covalently coated with folic acid (FA) to create a unique antisense oligonucleotide (ASO) delivery system. LOR-2501, a 20-mer phosphorothioate ASO targeting the ribonucleotide reductase R1 subunit, was intended to be delivered into tumor cells via this technique. The complexes were prepared at an optimized charge ratio of FA:PEI:LOR-2501 = 15:6:1, resulting in nanoparticles with a zeta potential of approximately +15.73 mV before FA addition, and near-neutral to slightly negative values after FA coating. Both folate receptor-positive and -negative cancer cells (e.g., HeLa, KB, A549, SK-HEP-1) exhibited markedly increased cellular uptake and gene silencing effectiveness in response to the FA/PEI-OA/LOR-2501 complexes. Flow cytometry confirmed that uptake in A549 cells was primarily through clathrin-mediated endocytosis, with sucrose inhibiting uptake by 80.5%. Despite the presence of FA, it was discovered that the increased absorption was unrelated to folate receptor-mediated endocytosis. Instead, clathrin-mediated endocytosis was the primary mechanism by which the complexes entered cells. The delivery demonstrated its therapeutic promise, inducing R1 mRNA downregulation by 51.7% in HeLa cells and 45.57% in A549 cells, and corresponding protein level reductions of 59.95% and 63.6%, respectively. This study emphasizes how FA improves the functionality of PEI-based gene delivery systems in a non-receptor-dependent manner.

Jin et.al. [18] evaluated PEI–PCL–PEI/PEG–PCL nanoparticles as siRNA carriers for gene therapy and demonstrated efficient siRNA encapsulation (~40%) at N/P ratios above 5, as confirmed by a SYBR Gold assay. Among the tested formulations, the one containing 2 µL PEG–PCL at N/P 7 exhibited superior cellular uptake, surpassing hyperbranched PEI (25 kDa) controls, as confirmed by flow cytometry. GFP knockdown was ~40% without chloroquine, rising to ~70% and ~95% with 50 and 100 µM chloroquine, respectively. This enhancement confirmed the carrier’s effective intracellular release upon endosomal disruption. Furthermore, MTT assays confirmed that the PEGylated nanoparticles exhibited lower cytotoxicity with IC_50_ values ranging from 37.40 to 40.13 µg/mL as compared to 13.58 µg/mL from the hyperbranched PEI (25 kDa), with IC_50_ values referring to the total nanoparticle mass, including both polymer and siRNA. This suggests that the reduction observed in cytotoxicity is due to enhanced biocompatibility.

Park et al. [17] created hyaluronic acid (HA)-shielded polyethylenimine (PEI)/plasmid DNA (pDNA) nanogels targeted at human mesenchymal stem cells (hMSCs) in order to create a receptor-mediated gene delivery system. Upregulation of CD44 receptors from hMSCs, which are HA’s natural ligands, served as the main justification. Their work demonstrated the ability to reconcile the reduction in PEI-associated cytotoxicity with the enhancement of cellular absorption by electrostatically coating PEI/pDNA complexes with HA. PEI:HA (1:10) showed up to 98% internalization in hMSCs after 6 h. Anti-CD44 antibodies reduced uptake from 96.32% to 35.44%, showing CD44-specific endocytosis. Enhanced chondrogenic differentiation was demonstrated by functional transfection with a pEGFP-SOX9 plasmid, which supported histological labeling and elevated the expression of SOX9 (~2.3-fold), Aggrecan (~2.6-fold), and COMP (~2.1-fold) at the mRNA and protein levels. This work shows that HA shielding makes receptor-targeted, low-toxicity, and effective gene delivery possible, which is very helpful for stem cell-based regenerative therapies.

Wang et al. [62] developed a ternary gene complex composed of pDNA, a PEI- lithocholic acid (LCA) conjugate (lp), and hyaluronic acid (HA), optimized for size, stability, and transfection efficiency both in vitro and in vivo. Their high-lp-to-pDNA ratio complex (Hi-DlpH) showed enhanced stability and cellular uptake, and a ~5.7 higher in vitro transfection efficiency compared to a low-ratio counterpart (Lo-DlpH), particularly in smaller Huh7 tumors (<300 mm^3^). However, in larger tumors (>500 mm^3^), Hi-DlpH was less effective; gene expression was reduced by ~65%. Flow cytometry of dissociated tumor tissue revealed that ~60% of Hi-DlpH-positive cells were F4/80^+^ Tumor-Associated Macrophages (TAMs); in contrast, only ~20% were tumor cells, confirming that TAMs acted as a significant sink, reducing gene delivery efficacy.

To accomplish CD44-targeted gene delivery, Liang et al. [53] presented a self-assembled ternary complex system made up of pDNA, branching PEI, and hyaluronic acid–epigallocatechin gallate (HA–EGCG) conjugates. This design used a dual-functional strategy: EGCG, an antioxidant polyphenol from green tea, provided strong DNA binding by hydrogen bonding and π–π stacking, as well as nuclease inhibition to protect the payload, while HA provided targeting capabilities via CD44. Superior physicochemical stability was demonstrated by the ternary complexes, which were resistant to polyanion-induced dissociation at C/P ratios ≥ 5 and showed no detectable pDNA release at C/P = 10, and they protected DNA from DNase I degradation for at least 2 h at 37 °C. Even in medium containing serum, transfection efficacy was markedly enhanced in HCT-116 colorectal cancer cells, which exhibited high levels of CD44 expression, according to in vitro tests. Transfection efficiency reached 43.7 ± 1.5% at a C/P ratio of 0.5, compared to 19.0 ± 0.8% for binary PEI/pDNA complexes. Free HA or anti-CD44 antibody significantly reduced transfection to 17.2 ± 3.1%, indicating CD44-mediated internalization. Compared to binary pDNA/PEI or even conventional HA-coated ternary complexes, confocal imaging showed 41.9 ± 2.5% nuclear localization of pDNA. To overcome obstacles in gene delivery, particularly in tumor cells that are challenging to transfect, this approach took advantage of the synergy of multivalent DNA binding and receptor-targeting.

**Figure 10:**
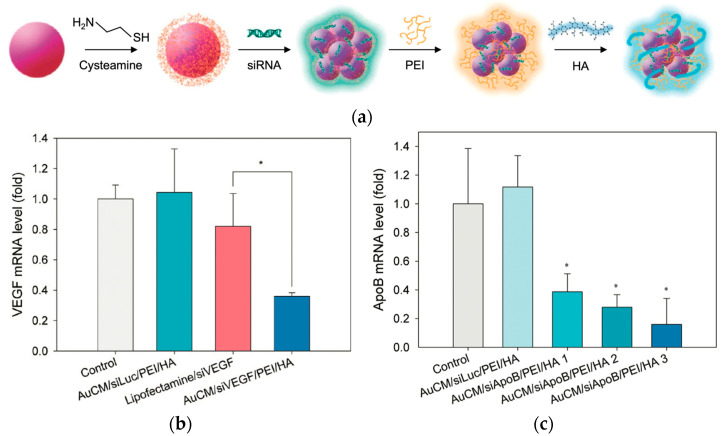
Nanoparticle complex for target-specific intracellular delivery of siRNA via HA receptor-mediated endocytosis. (**a**) Schematic illustration of the layer-by-layer assembly process used to prepare the AuCM/siRNA/PEI/HA nanocomplex. (**b**) VEGF gene silencing efficiency of the AuCM/siVEGF/PEI/HA complex in the presence of 50% serum, compared with the control groups: AuCM loaded with non-specific siRNA and the siVEGF/Lipofectamine complex. Data are presented as mean ± standard deviation (n = 3); *p* < 0.05. (**c**) ApoB mRNA expression levels following systemic delivery of target-specific gene silencing using three formulations of AuCM/siApoB/PEI/HA complexes at siRNA doses of 0.45, 0.90, and 1.8 nmol per mouse, compared to the control. Data are shown as mean ± standard deviation (n = 4); (*) *p* < 0.05 versus the control. Reproduced or adapted from [63], with permission from ACS Publications, 2025.

Gene silencing is a form of gene therapy that utilizes tools such as siRNA to suppress the expression of disease-causing genes at the mRNA level, thereby reducing the production of the corresponding proteins. Lee et al. [63] developed a cysteamine-modified gold nanoparticle from a (AuCM)/siRNA/PEI/hyaluronic acid (HA) complex using a layer-by-layer method for target-specific intracellular delivery of siRNA via HA receptor-mediated endocytosis (Figure 10). Each AuCM carried approximately 44 siRNA molecules, and the resulting nanocomplex had a hydrodynamic diameter of ~165.5 nm and a zeta potential of −12.1 mV. Analyses confirmed the effective cellular uptake of the complex by B16F1 cells through HA receptors, with cell viability remaining above 90% across all tested concentrations, indicating negligible cytotoxicity. PEI served as a key component for binding the negatively charged AuCM/siRNA via electrostatic interactions and promoted endosomal escape through the proton sponge effect, enabling effective cytoplasmic release. The authors also compared the use of PEI with the conventional transfection agent Lipofectamine 2000, emphasizing PEI’s robustness in complex biological environments. At equivalent siRNA doses (10 nM), the AuCM/PEI/HA complex achieved ~70% silencing of ApoB mRNA in vivo, compared to ~40% with Lipofectamine 2000. Additionally, VEGF knockdown reached 70–80% in the presence of 50% serum. MTT assays showed >90% cell viability for the AuCM/siRNA/PEI/HA complex, while free PEI (25 kDa) reduced viability to ~65% at the same concentration (20 μg/mL). HA coating mitigated PEI-induced toxicity. Biodistribution analysis revealed ~40% accumulation in liver tissue and ~17% in the spleen after systemic administration, demonstrating the platform’s potential for organ-targeted RNAi-based therapeutics.

Targeting ligands such as aptamers and hyaluronic acid (HA) further enhance tumor retention and reduce metastasis. Dehshahri et al. [64] enhanced PEI’s gene delivery by introducing hydrophobic alkyl chains to improve membrane interaction and cellular uptake, while incorporating oligoamines to restore primary amine density, thereby preserving DNA binding and endosomal escape via the proton sponge effect. The modified PEI demonstrated improved performance metrics: nanoparticles of ~75 nm in size, a zeta potential of +20–35 mV, and IL-12 gene expression levels up to 12-fold higher than naked DNA. Importantly, the cytotoxicity remained acceptable, with >70% viability at moderate N/P ratios, and toxicity only rising significantly at high N/P = 45 (viability ≈ 30%). These balanced enhancements support PEI’s safer, more effective use in non-viral gene therapy platforms.

A stable delivery system can reach target cells or tissues without being prematurely degraded, aggregated, or cleared from the bloodstream. Zhang et al. [16] proved that sodium alginate, when crosslinked with Ca^2+^, provided the optimal balance of biocompatibility, stability, and gene delivery efficiency, which makes it the best candidate for enhancing PEI-based gene therapy platforms. The stability of the complexes was confirmed by both in vitro and in vivo evaluations. The Ca^2+^/(Alg/PEI/DNA) nanoparticles with a weight ratio of 5:1:1 and 0.5 mM Ca^2^ showed an improved transfection efficiency of 32.4% in HeLa cells, which was 2.3-fold higher than PEI/DNA alone. Cellular uptake was also improved 2.2-fold, and cell viability was maintained at 86.1%, compared to 61.9% with PEI/DNA. In vivo studies in tumor-bearing mice revealed ~2.6-fold higher fluorescence intensity in tumors and lower distribution in the liver (by 48%) and kidneys (by 55%), attributed to the nanoparticles’ enhanced stability and EPR effect. Overall, this system shows strong potential for targeted gene delivery and tumor therapy.

### 4.2. Obstacles for Transitioning to Clinical Use

In preclinical experiments, PEI demonstrated exceptional nucleic acid condensation and transfection efficiency, indicating its significant promise as a non-viral gene delivery vector. However, numerous concerns have hindered its medicinal application, such as cytotoxicity, inadequate biocompatibility, and ineffective endosomal escape. PEI polyplexes also have short circulation periods and poor systemic stability, which cause them to be quickly removed from the bloodstream. Clinical trials are rare and have frequently terminated early because of safety, effectiveness, or recruitment concerns, despite promising results in animal models. These restrictions draw attention to the discrepancy between clinical translation and preclinical success [65].

#### 4.2.1. High Cytotoxicity

PEI is a widely used cationic polymer in gene transfer/therapy protocols due to its ability to condense DNA and promote cellular uptake with high transfection efficiency both in vitro and in vivo. It is also important to note that PEI is also cytotoxic; its cytotoxicity remains a critical barrier to clinical translation. While earlier studies assessed cytotoxicity through general metabolic assays (e.g., MTT), the exact molecular mechanisms underlying this process were poorly understood.

Moghimi et al. [66] investigated and defined the molecular pathways and stages through which branched and linear PEI, as well as their DNA complexes, induce cell damage and death in clinically relevant human cell lines, specifically on A549 human epithelial lung carcinoma cells and primary human umbilical vein endothelial cells. They revealed that PEI induces cytotoxicity through a distinct two-stage mechanism. In the first stage, occurring within 30 min of exposure, PEI caused rapid plasma membrane disruption, evidenced by significant lactate dehydrogenase release and phosphatidylserine translocation, indicating early necrotic-like cell damage. In the second stage, observed approximately 24 h post-exposure, cells exhibited signs of mitochondria-dependent apoptosis, including loss of mitochondrial membrane potential, cytochrome c release, caspase-3 activation, and nuclear DNA fragmentation. Both branched and linear PEI, as well as their DNA complexes, triggered similar biphasic responses, with DNA complexes showing slightly reduced early membrane damage but still inducing apoptosis at later stages. These findings explain PEI’s known toxicity in gene therapy and highlight the need for safer, modified PEI derivatives in biomedical applications.

Gao et al. [67] performed a study with the target of clarifying the mechanism of PEI-induced cytotoxicity in kidney and liver cell lines and keeping the focus on autography, and their findings concluded that autophagy plays a causative role in PEI cytotoxicity in kidney and liver cells. Unlike some earlier studies that suggested autophagy could be protective, this work clearly showed that excessive autophagy exacerbated cell damage, particularly through lysosomal and mitochondrial injury. The findings suggested that designing PEI-based vectors that avoided the endolysosomal pathway could help to reduce toxicity and enhance safety for gene therapy applications.

Lin et al. [68] investigated this mechanism on the cytotoxicity of branched PEI (25 kDa) and poly(l-lysine) with a focus on the role of autophagy in modulating the cell death of HeLa cells and atg5 knockout mouse embryonic fibroblasts. It was demonstrated that PEI induced both necrosis and apoptosis in a dose- and time-dependent manner. Crucially, autophagy exhibited a protective function on PEI-induced cell death, as it significantly inhibited both the necrosis and apoptosis promoted by PEI.

#### 4.2.2. Lack of Biodegradability

One significant disadvantage of PEI-based nanoparticles is their general cationic charge, which causes cytotoxic effects due to cellular organelle damage and non-specific interactions with serum proteins and negatively charged components of non-targeted cells. Furthermore, PEI’s non-biodegradable nature hinders quick removal, which increases cellular toxicity [69]. This has essentially shifted the focus to designing PEI delivery systems that ensure biodegradability and prevent off-target interactions.

High-molecular-weight (HMW) PEI derivatives, such as 25 kDa branched PEI and 22 kDa linear PEI, have traditionally been regarded as benchmark carriers in polycation-based gene delivery systems. However, while low-molecular-weight (LMW) PEI is associated with reduced cytotoxicity, it often shows compromised transfection efficiency [67]. To address this challenge, often referred to as the “PEI dilemma”, two main strategies have been introduced. One approach involves crosslinking LMW PEI using bioreducible linkers, resulting in biodegradable polymeric assemblies that exhibit HMW-like properties, rather than having a true HMW [70]. In PEI systems, the combination of ester and disulfide crosslinkers enables phased intracellular breakdown. Disulfide bonds break down in the reducing cytoplasm, guaranteeing full DNA release, while ester bonds hydrolyze in the acidic endosomal environment, allowing for early unpacking. By preserving stability during cellular absorption and encouraging efficient intracellular disintegration, this sequential degradation improves transfection efficiency.

The other approach aims to enhance PEI’s biocompatibility by modifying it with polysaccharides or hydrophobic groups, which helps to shield the polymer’s cationic surface [71]. Crosslinked LMW PEI provides an adequate positive charge for nucleic acid condensation while maintaining low cytotoxicity. Moreover, the cleavage of biodegradable linkers between PEI units facilitates efficient unpacking of genetic material, an essential feature often hindered in non-biodegradable PEI systems [72], where a variety of bioreducible linkers have been developed for this purpose.

Many bioreducible linkers were introduced to be crosslinked with LMW PEI [65], mostly redox-sensitive linkers like esters [73,74], carbamate [75], and disulfides [71]. Others were pH-sensitive linkers like polyaspartate [76], hydrazones [77], ketals, and glutadialdehyde [78]. In addition, the shielding-of-cationic-charge approach by polysaccharides included coupling PEI with different biodegradable and biocompatible compounds like dextran [79], cyclodextrin [80], and starch [81], as well as chitosan [82,83], including *N*-octyl-*N*-quaternary chitosan [84] and *O*-carboxymethyl chitosan [85]. All these mentioned conjugates have proven improved biocompatibility while maintaining and sometimes showing better transfection efficiency.

#### 4.2.3. Non-Specific Interactions with Serum Components

PEI is very effective as a gene delivery factor but it faces significant limitations in systemic administration due to its non-specific interactions with blood components [65]. Upon intravenous injection, PEI polyplexes have been shown to bind strongly to erythrocytes, platelets, lymphocytes, and negatively charged plasma proteins such as albumin, IgM, fibrinogen, fibronectin, and complement factors C3, C4, and C5 [86,87,88]. These interactions are largely driven by PEI’s highly cationic nature. Notably, PEI induces morphological changes in red blood cells, transforming them into non-circulating echinocytes [86], although this effect does not typically result in hemolysis [89]. Additionally, PEI polyplexes can bind with platelets and lymphocytes without activating them [90], yet still promote dose-dependent platelet aggregation. The formation of polyplex–cell aggregates has been observed particularly in the lung vasculature, often involving CD11b-positive immune cells such as monocytes, granulocytes, NK cells, and CD8^+^ T lymphocytes [91].

These aggregates can result in vascular occlusion, embolism, and localized pulmonary transfection, which significantly reduces the safety and specificity of gene delivery [86]. Moreover, PEI’s interactions with complement proteins, particularly through the alternative complement activation pathway, can cause complement activation-related pseudo-allergy [92,93]. This effect is thought to be compounded by pulmonary macrophage activation, which results in the release of thromboxane A2, a potent mediator of vasoconstriction, bronchoconstriction, and pulmonary hypertension [92]. Collectively, these negative responses contribute to rapid clearance, poor biodistribution, and increased risk of cardiopulmonary complications, posing a significant barrier to the clinical translation of PEI-based systems for systemic gene therapy. Strategies such as PEGylation and other surface modifications are being explored to mitigate these effects and improve the biocompatibility of PEI polyplexes [94,95].

Gustafson et al. [96] examined the difficulties posed by the mononuclear phagocyte system’s (MPS) non-specific uptake of nanoparticles, highlighting the function of protein adsorption (also known as the “protein corona”) in facilitating quick removal from the bloodstream. It was observed that upon entering biological fluids, nanoparticles were rapidly coated by serum proteins, including albumin, immunoglobulins, and fibrinogen. This caused opsonization and effective recognition by macrophages in filtration organs like the lungs, liver, and spleen. This study made clear how surface characteristics, such as charge, hydrophobicity, and curvature, had a significant impact on how proteins bind with nanoparticle surfaces and how the immune system recognizes them. Positively charged nanoparticles showed enhanced absorption because of electrostatic interactions with serum constituents and cell membranes. As a result, instead of reaching their intended tissues, more than 95% of the given nanoparticles frequently aggregated in MPS organs. In addition, Wilhelm et al. [97] noted that these non-specific interactions could cause tissue toxicity, complement activation, and inflammatory reactions. They promoted surface engineering techniques, such as PEGylation, to increase the stealth of nanoparticles and extend their circulation time.

#### 4.2.4. Poor Systemic Stability and Short Circulation Time

PEI’s inherent cationic charge causes non-specific interactions with cellular constituents, opsonization, and subsequent clearance by cells of the mononuclear phagocyte system (MPS), which accounts for the short circulation time and adverse events seen with unshielded polyplexes in the bloodstream. In addition, particle characteristics including size and shape have a significant impact on the MPS’s ability to absorb nanoparticles [96,98].

Steric stabilization is the most widely used strategy to enhance systemic circulation that prevents non-specific interactions. In order to prevent non-specific interactions with serum components, a protective layer is then applied to the surface of the hydrophobic amino acids [94]. Glucocorticoids (GCs) [99], cholesterols [100], various fatty acids [101], poly sarcosine [102], and hydroxyethyl starch [103], are among others that can be used to sterically stabilize PEI polyplexes and other non-viral formulations.

#### 4.2.5. Limited Success in Clinical Trials

Despite promising preclinical results, PEI-based formulations for gene therapy have encountered significant challenges in clinical trials. Out of 38 trials conducted up until 2023, most are in Phase I or II, indicating that PEI-based gene therapies are still in the early stages of development. Key hurdles for success include issues with efficacy, safety, and recruitment. Of the 38 trials, 19 have been completed, 8 are still active, and 10 were terminated due to issues such as recruitment difficulties, lack of efficiency, or strategic business decisions. Nonetheless, 14 additional clinical trials have been started since 2018, accounting for 37% of all investigations, which demonstrates the continued high level of interest in PEI-based treatments. With an emphasis on plasmid DNA delivery, these trials mostly use formulations utilizing commercially available 22 kDa linear PEI and are investigating ways to improve its stability for clinical application [65].

## 5. Conclusions

This review aims to provide an overview of polyethylenimine (PEI), regarding its synthesis, processing and structure–property relationships, and biomedical applications in drug and gene delivery. In recent decades, PEI has been demonstrated as a highly tunable and cationic polymer for its unique molecular architecture that exhibits an exceptional binding capacity with biomolecules as well as an ease of modifications. Linear and branched PEIs have shown distinct advantages in drug and gene delivery. Its capability to form nanoparticles, fibers, films, hydrogels, and many other drug carrier formats makes PEI adaptable to a wide range of delivery routes and biomedical demands.

Although PEI’s applications are widely accepted, the use of PEI in drug and gene delivery still faces critical limitations, hindering its use in clinical trials. These concerns include cytotoxicity, non-degradability, and systemic instability under physiological conditions. To overcome these challenges, modifications of PEI using chemicals, copolymer formation, and surface engineering enable the future use of PEI. Some of the recent trends show the emergence of responsive, biodegradable PEI-based systems capable of site-specific delivery, gene transfection with minimal off-target effects, and enhanced biocompatibility. Several important future directions of PEI for drug and gene delivery are itemized as follows:

(1) Advancing PEI’s synthesis and structural tuning, such as gaining exact control over its molecular weight, branching degree, and grafting density, will be essential to balance its activity and safety. This can be carried out through controlled ring-opening polymerization of 2-oxazolines for LPEI and temperature- or pH-regulated aziridine polymerization for BPEI, both influencing transfection efficiency and cytotoxicity.
(2) Utilizing multifunctional PEI composites has demonstrated great potential for intelligent drug and gene delivery, particularly for those that incorporate targeting ligands, biodegradable linkers, or stimuli-responsive moieties.
(3) Managing PEI’s advantages by extending its application beyond drug and gene delivery to related fields, including bioimaging, diagnostics, and environmental cleanup.
(4) Developing new and additional strategies to improve PEI’s long-term toxicity, metabolic destiny, and immunogenicity to gain regulatory approval that translates basic fundamental research to clinical use.

Overall, PEI-based drug carriers are expected to be the frontrunner for drug/gene delivery and gene therapy. Their ease of processing and capability to manipulate structural features, such as crosslinking strategies, biocompatibility, and functionalization, as well as their biomedical integration, demonstrate the strengths of PEI-based drug carriers in drug and gene delivery.

## Figures and Tables

**Table 1:** Comparative performance of LPEI and BPEI across gene delivery, drug delivery, and co-delivery applications.

Applications	LPEI	BPEI	Ref.
Gene Delivery	Lower cytotoxicity; more controllable transfection; favorable for in vivo applications	Higher transfection efficiency due to strong proton sponge effect but with cytotoxicity	[16,17,18,19]
Drug Delivery	Forms stable nanocomplexes with tunable release; lower toxicity	High drug loading due to dense amine content; stronger interactions	[20,21,22]
Co-Delivery (Gene + Drug)	Enables dual delivery of nucleic acids and hydrophobic drugs with reduced toxicity; effective for in vivo applications with PEGylation or FA-targeting	Higher loading efficiency and strong complexation; often used with ligands or PEG to reduce cytotoxicity	[22,23,24]

**Table 2:** Comparative evaluation of PEI-based systems, showing the relationship between polymer molecular weight, transfection efficiency, and cytotoxicity.

PEI Form	PEI MW	Transfection Efficiency	Cell Viability	Remarks	Ref.	
LMW PEI	1.8 kDa	~20–30%	>95% @ 10 µg/mL	Minimal cytotoxicity but ineffective transfection	[18]	
BPEI (25 kDa)	25 kDa	~70–95%	~60% @ 10 µg/mL	Highly effective but significantly toxic; poor safety profile for therapeutic applications	[18]	
PEI–PCL–PEI + PEG–PCL (2 µL added)	5–4 kDa	~70–80%	~85–90% @ 10 µg/mL	High performance, enhanced uptake and reduced toxicity due to PEG shielding, and improved stability	[18]	
Moderately crosslinked LPEI (x-LPEI-2)	~22 kDa	~70–80%	~90% @ 20 µg/mL	Retained high transfection efficiency while dramatically reducing cytotoxicity	[19]	
BPEI	25 kDa	~40–50%	~50–60% @ 20 µg/mL	Lower transfection efficiency and higher toxicity compared to crosslinked LPEI (xLPEI-2)	[19]	
Free PEI	10 kDa	~40–50%	~50–60% @ 20 µg/mL	Unmodified PEI; effective but highly toxic	[41]	
PPS NPs (PEI + PDA + siRNA)	10 kDa	~55–60%	~65–70% @ 20 µg/mL	Intermediate formulation without MnO_2_/FA, moderately safe, and moderately effective	[41]	
PPSM NPs (PEI + PDA + siRNA + MnO_2_ + FA)	10 kDa	~70–80%	~80–90% @ 20 µg/mL	Optimized nanoparticle with FA and MnO_2_; best balance of efficiency and safety	[41]	
CNOC-PEI-PEG	600 Da	N/A	>90% @ ≤100 µg/mL	Very low toxicity due to low-MW PEI	[42]	
BPEI	25 kDa	~60–70%	~40–50% @ 10 µg/mL	Strong condensation and delivery, but high cytotoxicity at >5 µg/mL	[45]	
LPEI	2.5 kDa	~20–30%	~80–90% @ 10 µg/mL	Poor gene delivery alone; considered safer but less potent	[45]	

## References

[1] Wang A.Z., Langer R., Farokhzad O.C. (2012). Nanoparticle delivery of cancer drugs. Annu. Rev. Med..

[2] Mura S., Nicolas J., Couvreur P. (2013). Stimuli-responsive nanocarriers for drug delivery. Nat. Mater..

[3] Zhang J., Cao Q., Li S., Lu X., Zhao Y., Guan J.-S., Chen J.-C., Wu Q., Chen G.-Q. (2013). Hydroxybutyrate methyl ester as a potential drug against Alzheimer’s disease via mitochondria protection mechanism. Biomaterials.

[4] Dobrovolskaia M.A., McNeil S.E. (2007). Immunological properties of engineered nanomaterials. Nat. Nanotechnol..

[5] Blanco E., Shen H., Ferrari M. (2015). Principles of nanoparticle design for overcoming biological barriers to drug delivery. Nat. Biotechnol..

[6] Yin H., Kanasty R.L., Eltoukhy A.A., Vegas A.J., Dorkin J.R., Anderson D.G. (2014). Non-viral vectors for gene-based therapy. Nat. Rev. Genet..

[7] Gilleron J., Querbes W., Zeigerer A., Borodovsky A., Marsico G., Schubert U., Manygoats K., Seifert S., Andree C., Stöter M. (2013). Image-based analysis of lipid nanoparticle–mediated siRNA delivery, intracellular trafficking and endosomal escape. Nat. Biotechnol..

[8] Lv H., Zhang S., Wang B., Cui S., Yan J. (2006). Toxicity of cationic lipids and cationic polymers in gene delivery. J. Control. Release.

[9] Pack D.W., Hoffman A.S., Pun S., Stayton P.S. (2005). Design and development of polymers for gene delivery. Nat. Rev. Drug Discov..

[10] Boussif O., Lezoualc’ht F., Zanta M.A., Mergnyt D., Schermant D., Demeneixt B., Behr J.-P. (1995). A versatile vector for gene and oligonucleotide transfer into cells in culture and in vivo: Polyethylenimine. Proc. Natl. Acad. Sci. USA.

[11] Lungwitz U., Breunig M., Blunk T., Göpferich A. (2005). Polyethylenimine-based non-viral gene delivery systems. Eur. J. Pharm. Biopharm..

[12] Fattahi N., Gorgannezhad L., Masoule S.F., Babanejad N., Ramazani A., Raoufi M., Sharifikolouei E., Foroumadi A., Khoobi M. (2024). PEI-based functional materials: Fabrication techniques, properties, and biomedical applications. Adv. Colloid Interface Sci..

[13] Mees M.A., Hoogenboom R. (2018). Full and partial hydrolysis of poly(2-oxazoline)s and the subsequent post-polymerization modification of the resulting polyethylenimine (co)polymers. Polym. Chem..

[14] Zhao C., Zhou B. (2023). Polyethyleneimine-based drug delivery systems for cancer theranostics. J. Funct. Biomater..

[15] Al-Absi A.A., Ogungbenro A.E., Benneker A.M., Mahinpey N. (2024). Review of polyethylenimine through ring-opening polymerization reactions and its application in CO_2_ capture. J. Environ. Chem. Eng..

[16] Zhang Y., Lin L., Liu L., Liu F., Maruyama A., Tian H., Chen X. (2018). Ionic-crosslinked polysaccharide/PEI/DNA nanoparticles for stabilized gene delivery. Carbohydr. Polym..

[17] Park J.S., Yi S.W., Kim H.J., Park K.-H. (2016). Receptor-mediated gene delivery into human mesenchymal stem cells using hyaluronic acid-shielded polyethylenimine/pDNA nanogels. Carbohydr. Polym..

[18] Jin Y., Adams F., Möller J., Isert L., Zimmermann C.M., Keul D., Merkel O.M. (2023). Synthesis and application of low molecular weight PEI-based copolymers for siRNA delivery with smart polymer blends. Macromol. Biosci..

[19] Bonner D.K., Zhao X., Buss H., Langer R., Hammond P.T. (2013). Crosslinked linear polyethylenimine enhances delivery of DNA to the cytoplasm. J. Control. Release.

[20] Tamaddon A., Abolmaali S.S., Yousefi G., Javidnia K., Dinarvand R. (2014). Sequential optimization of methotrexate encapsulation in micellar nano-networks of polyethyleneimine ionomer containing redox-sensitive cross-links. Int. J. Nanomed..

[21] Ratanajanchai M., Soodvilai S., Pimpha N., Sunintaboon P. (2014). Polyethylenimine-immobilized core–shell nanoparticles: Synthesis, characterization, and biocompatibility test. Mater. Sci. Eng. C.

[22] Zhou B., Zhao L., Shen M., Zhao J., Shi X. (2017). A multifunctional polyethylenimine-based nanoplatform for targeted anticancer drug delivery to tumors in vivo. J. Mater. Chem. B.

[23] Seo S.-J., Lee S.-Y., Choi S.-J., Kim H.-W. (2015). Tumor-targeting co-delivery of drug and gene from tempera-ture-triggered micelles. Macromol. Biosci..

[24] Liu C., Liu F., Feng L., Li M., Zhang J., Zhang N. (2013). The targeted co-delivery of DNA and doxorubicin to tumor cells via multifunctional PEI-PEG based nanoparticles. Biomaterials.

[25] Fernandes J.C., Qiu X., Winnik F.M., Benderdour M., Zhang X., Dai K., Shi Q. (2013). Linear polyethylenimine produced by partial acid hydrolysis of poly(2-ethyl-2-oxazoline) for DNA and siRNA delivery in vitro. Int. J. Nanomed..

[26] Tauhardt L., Kempe K., Knop K., Altuntaş E., Jäger M., Schubert S., Fischer D., Schubert U.S. (2011). Linear polyethyleneimine: Optimized synthesis and characterization—On the way to “Pharmagrade” batches. Macromol. Chem. Phys..

[27] Dick C.R., Ham G.E. (1970). Characterization of Polyethylenimine. J. Macromol. Sci. Part A—Chem..

[28] Brodie C.N., Goodfellow A.S., Andrews M.J., Owen A.E., Bühl M., Kumar A. (2024). Direct synthesis of partially ethoxylated branched polyethylenimine from ethanolamine. Nat. Commun..

[29] Zhang W., Chen D., Wang X., Xie X. (2022). Insight into the synthesis of branched polyethylenimine from 2-haloethylamine via a one-pot two-stage process. Polymer.

[30] Gleede T., Reisman L., Rieger E., Mbarushimana P.C., Rupar P.A., Wurm F.R. (2019). Aziridines and azetidines: Building blocks for polyamines by anionic and cationic ring-opening polymerization. Polym. Chem..

[31] Zou Y., Li D., Shen M., Shi X. (2019). Polyethylenimine-based nanogels for biomedical applications. Macromol. Biosci..

[32] Sahiner N., Sagbas S., Sahiner M., Demirci S. (2016). Degradable tannic acid/polyethyleneimine polyplex particles with highly antioxidant and antimicrobial effects. Polym. Degrad. Stab..

[33] Peng H., Rübsam K., Jakob F., Schwaneberg U., Pich A. (2016). Tunable enzymatic activity and enhanced stability of cellulase immobilized in biohybrid nanogels. Biomacromolecules.

[34] Sun W., Yang J., Zhu J., Zhou Y., Li J., Zhu X., Shen M., Zhang G., Shi X. (2016). Immobilization of iron oxide nanoparticles within alginate nanogels for enhanced MR imaging applications. Biomater. Sci..

[35] Wu Y., Jiang L., Dong Z., Chen S., Yu X.-Y., Tang S. (2021). Intracellular delivery of proteins into living cells by low-molecular-weight polyethyleneimine. Int. J. Nanomed..

[36] Pandey A.P., Sawant K.K. (2016). Polyethylenimine: A versatile, multifunctional non-viral vector for nucleic acid delivery. Mater. Sci. Eng. C.

[37] Bronstein L.M., Sidorov S.N., Gourkova A.Y., Valetsky P.M., Hartmann J., Breulmann M., Cölfen H., Antonietti M. (1998). Interaction of metal compounds with ‘double-hydrophilic’ block copolymers in aqueous medium and metal colloid formation. Inorg. Chim. Acta.

[38] Hu R., Wang L., Xu S., Lu Y., Zhou S. (2024). Silica nanospheres-encapsulated polymer ligands-bound Pd nanoparticles as highly efficient and selective catalysts for semi-hydrogenations of alkynes. Microporous Mesoporous Mater..

[39] Sidorov S.N., Bronstein L.M., Valetsky P.M., Hartmann J., Cölfen H., Schnablegger H., Antonietti M. (1999). Stabilization of metal nanoparticles in aqueous medium by polyethyleneoxide–polyethyleneimine block copolymers. J. Colloid Interface Sci..

[40] Hall A., Lächelt U., Bartek J., Wagner E., Moghimi S.M. (2017). Polyplex evolution: Understanding biology, optimizing performance. Mol. Ther..

[41] Li J., Yu X., Shi X., Shen M. (2022). Cancer nanomedicine based on polyethylenimine-mediated multifunctional nanosystems. Prog. Mater. Sci..

[42] Sun W., Zhang X., Jia H.R., Zhu Y.X., Guo Y., Gao G., Li Y.H., Wu F.G. (2019). Water-dispersible candle soot–derived carbon nano-onion clusters for imaging-guided photothermal cancer therapy. Small.

[43] Sun X., Dong S., Wang E. (2004). One-step synthesis and characterization of polyelectrolyte-protected gold nanoparticles through a thermal process. Polymer.

[44] Gorejová R., Podrojková N., Sisáková K., Shepa J., Shepa I., Kovalčíková A., Šišoláková I., Kaľavský F., Oriňaková R. (2022). Interaction of thin polyethyleneimine layer with the iron surface and its effect on the electrochemical behavior. Sci. Rep..

[45] Khanam N., Mikoryak C., Draper R.K., Balkus K.J. (2007). Electrospun linear polyethyleneimine scaffolds for cell growth. Acta Biomater..

[46] Xu X., Zhang J.-F., Fan Y. (2010). Fabrication of cross-linked polyethyleneimine microfibers by reactive electrospinning within situ photo-cross-linking by UV radiation. Biomacromolecules.

[47] Englert C., Tauhardt L., Hartlieb M., Kempe K., Gottschaldt M., Schubert U.S. (2014). Linear poly(ethylene imine)-based hydrogels for effective binding and release of DNA. Biomacromolecules.

[48] Chang C.-W., Ho H.-O., Lo Y.-J., Lee S.-Y., Yang Y.-R., Sheu M.-T. (2012). Development of swellable local implants of a polyethyleneimine-poly(vinyl pyrrolidone) (PEI-PVP) hydrogel as a socket filler. J. Biomater. Sci. Polym. Ed..

[49] Avais M., Chattopadhyay S. (2019). Waterborne pH responsive hydrogels: Synthesis, characterization and selective pH responsive behavior around physiological pH. Polymer.

[50] Wu M.-B., Fan X.-L., Yang H.-C., Yang J., Zhu M.-M., Ren K.-F., Ji J., Xu Z.-K. (2019). Ultrafast formation of pyrogallol/polyethyleneimine nanofilms for aqueous and organic nanofiltration. J. Membr. Sci..

[51] Wen H., Liu Z., Lu Z., Yang Y., Chen J.P. (2025). High-performance PEI-based nanofiltration membrane by MXene-regulated interfacial polymerization reaction: Design, fabrication and testing. J. Membr. Sci..

[52] Yang S., Yang X., Liu Y., Zheng B., Meng L., Lee R.J., Xie J., Teng L. (2015). Non-covalent complexes of folic acid and oleic acid conjugated polyethylenimine: An efficient vehicle for antisense oligonucleotide delivery. Colloids Surf. B.

[53] Ding G.-B., Meng X., Yang P., Li B., Stauber R.H., Li Z. (2020). Integration of Polylactide into Polyethylenimine facilitates the safe and effective intracellular siRNA delivery. Polymers.

[54] Pandi J.S., Pavadai P., Babkiewicz E., Panneerselvam T., Maszczyk P., Sankaranarayanan M., Mohan R., Kunjiappan S. (2025). Targeted delivery of thymoquinone-encapsulated polyethyleneimine/poly (lactic acid) nanoparticles into breast cancer cells. Res. Sq..

[55] Xiao Y., Fan Y., Tu W., Ning Y., Zhu M., Liu Y., Shi X. (2021). Multifunctional PLGA microfibrous rings enable MR imaging-guided tumor chemotherapy and metastasis inhibition through prevention of circulating tumor cell shedding. Nano Today.

[56] Zhou B., Xiong Z., Zhu J., Shen M., Tang G., Peng C., Shi X. (2016). PEGylated polyethylenimine-entrapped gold nanoparticles loaded with Gadolinium for dual-mode Ct/Mr imaging applications. Nanomedicine.

[57] Duncan R., Ringsdorf H., Satchi-Fainaro R. (2006). Polymer therapeutics—Polymers as drugs, drug and protein conjugates and gene delivery systems: Past, present and future opportunities. J. Drug Target..

[58] Kamruzzaman Selim K.M., Ha Y.-S., Kim S.-J., Chang Y., Kim T.-J., Lee G., Kang I.-K. (2007). Surface modification of magnetite nanoparticles using lactobionic acid and their interaction with hepatocytes. Biomaterials.

[59] Huang Y., Zhan M., Shen M., Zhang L., Shi X. (2023). Electrospun short fibers: A new platform for cancer nanomedicine applications. Explor. Drug Sci..

[60] Ma Y., Li D., Xiao Y., Ouyang Z., Shen M., Shi X. (2021). LDH-doped electrospun short fibers enable dual drug loading and multistage release for chemotherapy of drug-resistant cancer cells. New J. Chem..

[61] de Souza A.P., Bastos A.P., da Fonseca F.N., Pandolfi J.R., Costamilan C.A.D.V.L.R., Marques M.G. (2024). Polyethyleneimine-mediated gene transfection in porcine fetal fibroblasts. Anim. Reprod..

[62] Wang J., Meng F., Kim B.K., Ke X., Yeo Y. (2019). In-vitro and in-vivo difference in gene delivery by lithocholic acid-polyethyleneimine conjugate. Biomaterials.

[63] Lee M.Y., Park S.J., Park K., Kim K.S., Lee H., Hahn S.K. (2011). Target-specific gene silencing of layer-by-layer assembled gold-cysteamine/siRNA/PEI/HA nanocomplex. ACS Nano.

[64] Dehshahri A., Alhashemi S.H., Jamshidzadeh A., Sabahi Z., Samani S.M., Sadeghpour H., Mohazabieh E., Fadaei M. (2013). Comparison of the effectiveness of polyethylenimine, polyamidoamine and chitosan in transferring plasmid encoding interleukin-12 gene into hepatocytes. Macromol. Res..

[65] Casper J., Schenk S.H., Parhizkar E., Detampel P., Dehshahri A., Huwyler J. (2023). Polyethylenimine (PEI) in gene therapy: Current status and clinical applications. J. Control. Release.

[66] Moghimi S.M., Symonds P., Murray J.C., Hunter A.C., Debska G., Szewczyk A. (2005). A two-stage poly(ethylenimine)-mediated cytotoxicity: Implications for gene transfer/therapy. Mol. Ther..

[67] Gao X., Yao L., Song Q., Zhu L., Xia Z., Xia H., Jiang X., Chen J., Chen H. (2011). The association of autophagy with polyethylenimine-induced cytotoxicity in nephritic and hepatic cell lines. Biomaterials.

[68] Lin C.W., Jan M.S., Kuo J.H.S., Hsu L.J., Lin Y.S. (2012). Protective role of autophagy in branched polyethylenimine (25K)- and poly(L-lysine) (30–70K)-induced cell death. Eur. J. Pharm. Sci..

[69] Jiang H.-L., Islam M.A., Xing L., Firdous J., Cao W., He Y.-J., Zhu Y., Cho K.-H., Li H.-S., Cho C.-S. (2017). Degradable polyethylenimine-based gene carriers for cancer therapy. Top. Curr. Chem..

[70] Nouri F., Sadeghpour H., Heidari R., Dehshahri A. (2017). Preparation, characterization, and transfection efficiency of low molecular weight polyethylenimine-based nanoparticles for delivery of the plasmid encoding CD200 gene. Int. J. Nanomed..

[71] Yu H., Russ V., Wagner E. (2009). Influence of the molecular weight of bioreducible oligoethylenimine conjugates on the polyplex transfection properties. AAPS J..

[72] Meng F., Hennink W.E., Zhong Z. (2009). Reduction-sensitive polymers and bioconjugates for biomedical applications. Biomaterials.

[73] Forrest M.L., Koerber J.T., Pack D.W. (2003). A degradable polyethylenimine derivative with low toxicity for highly efficient gene delivery. Bioconjug. Chem..

[74] Russ V., Elfberg H., Thoma C., Kloeckner J., Ogris M., Wagner E. (2008). Novel degradable oligoethylenimine acrylate ester-based pseudodendrimers for in vitro and in vivo gene transfer. Gene Ther..

[75] Wang Y.Q., Su J., Wu F., Lu P., Yuan L.F., Yuan W.E., Sheng J., Jin T. (2012). Biscarbamate cross-linked polyethylenimine derivative with low molecular weight, low cytotoxicity, and high efficiency for gene delivery. Int. J. Nanomed..

[76] Zhang G., Liu J., Yang Q., Zhuo R., Jiang X. (2012). Disulfide-containing brushed polyethylenimine derivative synthesized by click chemistry for nonviral gene delivery. Bioconjug. Chem..

[77] Fang G., Zeng F., Yu C., Wu S. (2014). Low molecular weight PEIs modified by hydrazone-based crosslinker and betaine as improved gene carriers. Colloids Surf. B.

[78] Kim Y.H., Park J.H., Lee M., Kim Y.-H., Park T.G., Kim S.W. (2005). Polyethylenimine with acid-labile linkages as a biodegradable gene carrier. J. Control. Release.

[79] Ochrimenko S., Vollrath A., Tauhardt L., Kempe K., Schubert S., Schubert U.S., Fischer D. (2014). Dextran-graft-linear poly(ethylene imine)s for gene delivery: Importance of the linking strategy. Carbohydr. Polym..

[80] Xie B., Peng J., Wang S., Zhang X., Nie H. (2016). Investigation of the sequential actions of doxorubicin and p53 on tumor cell growth via branched polyethylenimine-β-cyclodextrin conjugates. Ann. Biomed. Eng..

[81] Yamada H., Loretz B., Lehr C.-M. (2014). Design of starch-*graft*-PEI polymers: An effective and biodegradable gene delivery platform. Biomacromolecules.

[82] Yue J., Wu J., Liu D., Zhao X., Lu W.W. (2015). BMP2 gene delivery to bone mesenchymal stem cell by chitosan-g-PEI nonviral vector. Nanoscale Res. Lett..

[83] Lee Y.H., Park H.I., Choi J.S. (2016). Novel glycol chitosan-based polymeric gene carrier synthesized by a Michael addition reaction with low molecular weight polyethylenimine. Carbohydr. Polym..

[84] Liu K., Hui L., Qing Z., Kewu L., Manman Z., Wenfang Z., Yuan M. (2014). Coupling of a bifunctional peptide R13 to OTMCS-PEI copolymer as a gene vector increases transfection efficiency and tumor targeting. Int. J. Nanomed..

[85] Nam J.-P., Nah J.-W. (2016). Target gene delivery from targeting ligand conjugated chitosan–PEI copolymer for cancer therapy. Carbohydr. Polym..

[86] Zhong D., Jiao Y., Zhang Y., Zhang W., Li N., Zuo Q., Wang Q., Xue W., Liu Z. (2013). Effects of the gene carrier polyethyleneimines on structure and function of blood components. Biomaterials.

[87] Ogris M., Brunner S., Schüller S., Kircheis R., Wagner E. (1999). PEGylated DNA/transferrin–PEI complexes: Reduced interaction with blood components, extended circulation in blood and potential for systemic gene delivery. Gene Ther..

[88] Toy R., Pradhan P., Ramesh V., Di Paolo N.C., Lash B., Liu J., Blanchard E.L., Pinelli C.J., Santangelo P.J., Shayakhmetov D.M. (2019). Modification of primary amines to higher order amines reduces in vivo hematological and immunotoxicity of cationic nanocarriers through TLR4 and complement pathways. Biomaterials.

[89] Mehrizi T.Z. (2021). Hemocompatibility and hemolytic effects of functionalized nanoparticles on red blood cells: A recent review study. Nano.

[90] Goula D., Becker N., Lemkine G.F., Normandie P., Rodrigues J., Mantero S., Levi G., Demeneix B.A. (2000). Rapid crossing of the pulmonary endothelial barrier by polyethylenimine/DNA complexes. Gene Ther..

[91] Chollet P., Favrot M.C., Hurbin A., Coll J. (2002). Side-effects of a systemic injection of linear polyethylenimine–DNA complexes. J. Gene Med..

[92] Merkel O.M., Urbanics R., Bedőcs P., Rozsnyay Z., Rosivall L., Toth M., Kissel T., Szebeni J. (2011). In vitro and in vivo complement activation and related anaphylactic effects associated with polyethylenimine and polyethylenimine-graft-poly(ethylene glycol) block copolymers. Biomaterials.

[93] Chen F., Wang G., Griffin J.I., Brenneman B., Banda N.K., Holers V.M., Backos D.S., Wu L., Moghimi S.M., Simberg D. (2017). Complement proteins bind to nanoparticle protein corona and undergo dynamic exchange in vivo. Nat. Nanotechnol..

[94] Parhiz H., Hashemi M., Hatefi A., Shier W.T., Amel Farzad S., Ramezani M. (2013). Arginine-rich hydrophobic polyethylenimine: Potent agent with simple components for nucleic acid delivery. Int. J. Biol. Macromol..

[95] Lam A.K., Moen E.L., Pusavat J., Wouters C.L., Panlilio H., Ferrell M.J., Houck M.B., Glatzhofer D.T., Rice C.V. (2020). PEGylation of polyethylenimine lowers acute toxicity while retaining anti-biofilm and β-lactam potentiation properties against antibiotic-resistant pathogens. ACS Omega.

[96] Gustafson H.H., Holt-Casper D., Grainger D.W., Ghandehari H. (2015). Nanoparticle uptake: The phagocyte problem. Nano Today.

[97] Wilhelm S., Tavares A.J., Dai Q., Ohta S., Audet J., Dvorak H.F., Chan W.C.W. (2016). Analysis of nanoparticle delivery to tumours. Nat. Rev. Mater..

[98] Hoshyar N., Gray S., Han H., Bao G. (2016). The effect of nanoparticle size on in vivo pharmacokinetics and cellular interaction. Nanomedicine.

[99] Ma K., Hu M., Qi Y., Qiu L., Jin Y., Yu J., Li B. (2009). Structure–transfection activity relationships with glucocorticoid–polyethyl-enimine conjugate nuclear gene delivery systems. Biomaterials.

[100] Wu P., Luo X., Wu H., Zhang Q., Wang K., Sun M., Oupicky D. (2020). Combined hydrophobization of polyethylenimine with cholesterol and perfluorobutyrate improves siRNA delivery. Bioconjug. Chem..

[101] Dunn A.W., Kalinichenko V.V., Shi D. (2018). Highly efficient in vivo targeting of the pulmonary endothelium using novel modifications of polyethylenimine: An importance of charge. Adv. Healthc. Mater..

[102] Heller P., Birke A., Huesmann D., Weber B., Fischer K., Reske-Kunz A., Bros M., Barz M. (2014). Introducing PeptoPlexes: Polylysine-*block*-polysarcosine based polyplexes for transfection of HEK 293T cells. Macromol. Biosci..

[103] Noga M., Edinger D., Rödl W., Wagner E., Winter G., Besheer A. (2012). Controlled shielding and deshielding of gene delivery polyplexes using hydroxyethyl starch (HES) and alpha-amylase. J. Control. Release.

